# Phenotypic and histological analyses on the resistance of melon to *Phelipanche aegyptiaca*

**DOI:** 10.3389/fpls.2023.1070319

**Published:** 2023-03-24

**Authors:** Xiaolei Cao, Lifeng Xiao, Lu Zhang, Meixiu Chen, Pengxuan Bian, Qianqian Ma, Siyu Chen, Quanlong He, Xinli Ma, Zhaoqun Yao, Sifeng Zhao

**Affiliations:** ^1^ Key Laboratory of Oasis Agricultural Pest Management and Plant Protection Resources Utilization, Xinjiang Uygur Autonomous Region, Shihezi University, Shihezi, Xinjiang, China; ^2^ Key Laboratory of Special Fruits and Vegetables Cultivation Physiology and Germplasm Resources Utilization of Xinjiang Production and Construction Corps, Shihezi University, Shihezi, Xinjiang, China; ^3^ Hami Melon Research Center, Xinjiang Academy of Agricultural Science, Urumqi, Xinjiang, China

**Keywords:** melon, *Phelipanche aegyptiaca*, parasitic plants, histology, necrosis, resistance

## Abstract

Melon (*Cucumis melo* L.) is an economically important crop in Xinjiang, China, but its production is constrained by the parasitic plant *Phelipanche aegyptiaca* that attaches to the roots of many crops and causes severe stunting and loss of yield. Rhizotron, pot, and field experiments were employed to evaluate the resistance of 27 melon cultivars to *P. aegyptiaca*. Then, the resistant and susceptible cultivars were inoculated with *P. aegyptiaca* from six populations to assess their resistance stability and broad spectrum. Further microscopic and histological analyses were used to clarify the resistance phenotypes and histological structure. The results showed that Huangpi 9818 and KR1326 were more resistant to *P. aegyptiaca* compared to other cultivars in the rhizotron, pot, and field experiments. In addition, compared to the susceptible cultivar K1076, Huangpi 9818 and KR1326 showed broad-spectrum resistance to six *P. aegyptiaca* populations. These two resistant cultivars had lower *P. aegyptiaca* biomass and fewer and smaller *P. aegyptiaca* attachments on their roots compared to susceptible cultivar K1076. KR1326 (resistant) and K1076 (susceptible) were selected to further study resistance phenotypes and mechanisms. Germination-inducing activity of root exudates and microscopic analysis showed that the resistance in KR1326 was not related to low induction of *P. aegyptiaca* germination. The tubercles of parasite on KR1326 were observed slightly brown at 14 days after inoculation (DAI), the necrosis and arrest of parasite development occurred at 23 DAI. Histological analysis of necrosis tubercles showed that the endophyte of parasite had reached host central cylinder, connected with host xylem, and accumulation of secretions and callose were detected in neighbouring cells. We concluded that KR1326 is an important melon cultivar for *P. aegyptiaca* resistance that could be used to expand the genetic basis of cultivated muskmelon for resistance to the parasite.

## Introduction

Melon (*Cucumis melo* L.) is one of the most widely cultivated and economically important fruit crops in the world. In 2020, more than 184 million tons of melon were grown in China. Xinjiang melon accounts for over 50% of the total melon production in China, and the fruits are exported worldwide (Bin et al., 2022). *Philepanche aegyptiaca* is a serious threat that has caused severe yield losses of up to 20%–70% in melon crops in Xinjiang, China (Parker, 2009; Zhang et al., 2012; Yao et al., 2016). When the levels of infestation are so high that melon suffers 100% yield loss, farmers could be forced to abandon their fields or plant other crops that cannot be infected by *P. aegyptiaca* (Zhang et al., 2012).

*P. aegyptiaca* is an obligate root holoparasite weed that causes severe losses in the yield and quality of agricultural crops (Parker, 2009; Eizenberg and Goldwasser, 2018). Efficient control of this parasite is difficult due to its specific lifecycle. The seed of *P. aegyptiaca* can lie dormant for years or decades in the soil until chemical signals from a nearby host or nonhost root activate their germination (Nelson, 2021). Each germinated *P. aegyptiaca* seedling forms a radicle that in response to host-derived haustorium-inducing factors (HIFs) forms an organ called the haustorium (Furuta et al., 2021). Upon contact with a host root, the haustorium forms an intrusive cell that penetrates the host root cortex and endodermis to establish parasite–host xylem–xylem connections. Once a xylem bridge is established, parasites can quickly obtain water and nutrients from hosts for their own development (Pageau et al., 2003). However, *Orobanche* spp. can also acquire materials through phloem–phloem connections (Aly, 2013). Following the accumulation of metabolites, the parasite develops a tubercle, and the mature tubercle then develops a shoot that emerges above the ground and flowers to produce tiny seeds that can remain viable in the soil for many years (Eizenberg and Goldwasser, 2018). The major damage inflicted by *P. aegyptiaca* takes place underground, thereby limiting the use of chemical forms of control, making control extremely challenging. Several methods have been proposed for broomrape control in the field: alternating planting dates, soil solarization, soil fumigation, and chemical control, but all without unequivocal success (Fernández-Aparicio et al., 2012; Kannan et al., 2015; Dor et al., 2017). Breeding for resistance is commonly considered to be an effective, affordable, and environment-friendly component of an integrated control strategy (Eizenberg and Goldwasser, 2018). Host genetic resistance is also generally considered critical to successful integrated pest management programs (Joel, 2000; Goldwasser et al., 2001).

Useful levels of resistance have been found in several hosts against parasitic plants, for example in rice against *Striga hermonthica* (Gurney et al., 2006), in sorghum against *S. hermonthica* (Mbuvi et al., 2017), in cowpea against *Striga gesnerioides* (Su et al., 2020), in sunflower against *Orobanche cumana* (Fernández-Martínez et al., 2000; Echevarría-Zomeño et al., 2006; Duriez et al., 2019), in tomato against *P. aegyptiaca* (Bai et al., 2020), and faba bean (Díaz-Ruiz et al., 2010; Rubiales et al., 2014), chickpea (Rubiales et al., 2003), and vetch (Fernandez-Aparicio et al., 2009; González-Verdejo et al., 2021) against *Orobanche crenata.* The mechanisms of resistance to parasitic plants vary depending on the host species and cultivars (Mutuku et al., 2021; Jhu and Sinha, 2022). Based on whether the resistance mechanism functions before or after parasitic plants attach to their hosts, resistance responses can be classified as preattachment or postattachment resistance (Thorogood and Hiscock, 2010; Yoder and Scholes, 2010; Mutuku et al., 2021; Jhu and Sinha, 2022). Furthermore, preattachment resistance involves the production of lower levels of parasite germination stimulants (Rubiales, 2003; Gobena et al., 2017) and parasite haustorial inducing factors (Goyet et al., 2019). Other than simply avoiding the induction of parasite seed germination, some hosts have evolved to secrete toxic compounds to prevent seed germination or seedling development (Serghini et al., 2001; Echevarría-Zomeño et al., 2006). Postattachment resistance mechanisms include HRs, hormone signaling, cell wall reinforcement, and defensive secondary metabolite accumulation that prevents vascular continuity with the parasite after forming the haustorium (Jhu and Sinha, 2022). For example, some legumes resistant to *O. crenata* show necrosis of established tubercles. This is caused by lignification and occlusion of vascular tissue (accumulation of secretions) at the infection site (Pérez-de-Luque et al., 2005). These secretions appear to originate from parasite cells and flow through the apoplast to neighboring host tissues, occluding host vessel elements (Pérez-de-Luque et al., 2005; Pérez-de-Luque et al., 2006). Occlusion of vascular tissue and lignin deposition are common strategies deployed by many resistant host species (Thorogood and Hiscock, 2010; Jhu and Sinha, 2022). In the case of rice (Nipponbare), a cultivar resistant to *S. hermonthica* showed enhanced lignin deposition at the infection site, thereby inhibiting parasite development by lignin deposition and maintenance of the structural integrity of lignin polymers (Mutuku et al., 2019). Similar resistance responses also have been observed in the interaction between sunflower and *O. cumana.* Yang et al. (2017) also showed that the enzymes involved in lignin biosynthesis, including cinnamoyl alcohol dehydrogenase (CAD), ferulate-5-hydroxylase (F5H), and peroxidases, were highly accumulated in these resistant sunflower cultivars.

Our previous studies on melon have shown that cultivars exhibiting resistance may be an effective *P. aegyptiaca* control method. Peng et al. (2018) demonstrated that melon germplasm contains sources of postattachment resistance to *P. aegyptiaca*. For example, Jintianmi17 exhibits high resistance, with parasite development stagnating at the tubercle stage. Cao et al. (2020) reported that a wild accession PI 614391 and a cultivated accession Sekine exhibited higher tolerance to *P. aegyptiaca.* However, apart from the cultivars identified in these studies, few resistant melon cultivars have been found, and a good understanding of the resistance mechanism to *P. aegyptiaca* is still lacking. The objectives of the current study were: (1) to identify *P. aegyptiaca*-resistant melon cultivars in a series of rhizotron, pot, and field experiments, and (2) to characterize the phenotype of the resistance mechanisms.

## Materials and methods

### Plant materials

A total of 27 melon cultivars (**Supplementary Table 1**) were evaluated for resistance to *P. aegyptiaca*, together with details of the source of the seed and their origin. The melon cultivar Naxigan was used as a susceptible control for both rhizotron and pot assays (Peng et al., 2018). The origin and description of six *P. aegyptiaca* populations used in this study are shown in **Supplementary Table 2**. Total genomic DNA was extracted from the seeds of six broomrape populations, and the ribosomal protein S2 (*rps2*) and ribosomal DNA internal transcribed spacer (ITS) region were amplified by PCR using the primer pairs rps2F/rps2R, ITS1/ITS4 (Anderson and Cairney, 2004; Park et al., 2007). Based on phylogenetic analysis of plastid ITS and *rps2* (Schneeweiss et al., 2004; Park et al., 2007), we confirmed that our experimental species was *P. aegyptiaca* (**Supplementary Figure 1**). *O. cumana* seeds were collected from mature plants parasitizing sunflowers at 188 Regiment (87° 33′15″ E, 46° 6′ 9″ N, 500 m elevation), Xinjiang, China in 2019. *O. cumana* seeds were used as a control to detect the effects of root exudates of different melon cultivars on the germination of *P. aegyptiaca*.

### Rhizotron experiment

Rhizotron experiments were carried out under laboratory conditions as described by Labrousse et al. (2001) with minor modifications to observe the attachment and development of *P. aegyptiaca* throughout the study. Seeds per melon cultivar were sterilized with sodium hypochlorite solution (5% v / v) for 20 min, rinsed thrice in sterile deionized water, and germinated in petri dishes (15 cm diameter) on wet filter paper and kept in the dark at 30°C for 2–3 d (until the radicle reached 1 cm in length). Germinated seeds were grown in a 50-hole (5 × 5 × 5 cm) plastic cave dish containing vermiculite and were watered regularly with Hoagland nutrient solution. The seedlings were maintained in a growth chamber at 25°C with a day/night regime of 16/8 h and a 10 000 Lx light source. Briefly, 2-week-old seedlings were transferred to a rhizotron comprising a 15-cm petri dish filled with a sponge overlaid with glass fiber filter paper, and the plant roots were evenly spread out over the surface of the filter paper. To prevent exposure of host roots to direct light, the dishes containing plants were covered with aluminum foil. The rhizotrons were then kept in growth chambers at a temperature cycle of 28°C/25°C for a 16-h-light/8-h-dark cycle. After 1 week, the growing melon plants were inoculated with sterilized *P. aegyptiaca* seeds (Plot 2; approximately 400 seeds each) and were carefully placed next to the roots of each melon plant. The rhizotrons containing inoculated plants were placed back into the growth chambers and incubated under the same conditions until investigation.

Developmental stages of broomrape were recorded and classified at 35 d after inoculation (DAI) according to their stage of development using a scale from 0 to 9 (Westwood et al., 2012) with slight modifications: S0, *P. aegyptiaca* seeds were not germinated; S1, seed germination; S2, terminal haustorium formation (before the radical had firmly adhered to or penetrated the host roots); S3, broomrape had firmly adhered to or penetrated the host roots but had not formed a vascular connection; S4, broomrape had formed a vascular connection to the host roots and a tubercle had formed (before the development of secondary roots); S5, “spider” stage (tubercles with secondary roots) ≤ 1 cm; S6, sprout already visible remaining underground > 2 cm; S7, the start of the development of a spike; S8, flowering; S9, setting of seeds (Westwood et al., 2012).

### Pot evaluation

Pot experiments were carried out to evaluate the resistance of 27 melon cultivars in a greenhouse in 2017 and 2018. The seeds of *P. aegyptiaca* were collected from Plot 2. Germinated melon seeds were sown in 1.5-L pots containing a mixture of sand-vermiculite-compost (1:1:1, v:v:v) and *P. aegyptiaca* seeds (15 mg of *P. aegyptiaca* seeds per 1 kg of the substrate for the 2017 experiment and 30 mg of *P. aegyptiaca* seeds per 1 kg of the substrate for the 2018 experiment). Noninfected plants were grown and evaluated in parallel. The pots were arranged in a completely randomized design under greenhouse conditions. The plants were grown in a growth chamber at 25°C–30°C, with a day/night regime of 16/8h and with a 10 000 Lx light source (800–900 *μ*mol m^−2^ s^−1^). Pots were watered with about 150 ml of tap water every 5-7 days. Sixty days later, the plants were divided from the pots, and the roots were washed carefully in water. Attachments on host roots were counted and classified according to their stage of development using a scale from zero to nine as mentioned above.

The parasitism rate (percentage of melon plants infected by *P. aegyptiaca*) and the parasitic degree (the number of *P. aegyptiaca* per plant) were calculated. According to the resistance standards (**Supplementary Table 3**), the resistance levels of different melon varieties to *P. aegyptiaca* were identified (Peng et al., 2018).

In addition, morphological traits of melon (plant height (PH), stem diameter (SD), fresh (FGD) and dry weights of total aboveground parts (DGD), fresh (RFW), and dry weight of roots (RDW)) were measured. The fresh and dry weights of broomrape were also measured.

### Screening of melon cultivars for *P. aegyptiaca* resistance in the field

Field screening experiments were carried out to assess the behavior of melon cultivars selected for their partial resistance to *P. aegyptiaca*. A sketch of the field layout is provided in **Supplementary Figure 2**.

The *P. aegyptiaca* field trials were conducted at two sites; one was at the experimental station of the Shihezi University (May–Aug) in 2018, and the other was at Majiaping farm in Shihezi (May–Aug) in 2019 and 2021 (**Supplementary Table 4**). All field trials were replicated three times using a completely randomized block design. For control experiments, melon cultivars were simultaneously planted in *P. aegyptiaca*-free plots within the same locations. At the experimental station, each plot representing an individual cultivar measured 3 m × 3 m (9 m^2^) and contained one row of 10 melon plants with a plant distance of 0.3 m × 0.3 m (**Supplementary Figure 2**). At Majiaping, each plot measured 6.5 m × 3 m (19.5 m^2^) with two rows of 20 melon plants with the same plant distance as in the experimental station (**Supplementary Figure 2**). Plots were separated by one open row of 0.6 m to avoid neighbor effects and to allow easy access. From sowing onwards, each trial was regularly hand weeded (at least every 2–3 weeks) to remove all weeds other than *P. aegyptiaca*. The number of aboveground *P. aegyptiaca* plants in each plot was counted at 45, 60, and 80 d. At the maturity stage of melon, the fruit yields of noninfested and naturally infested fields by *P. aegyptiaca* were recorded.

### Resistance of different melon cultivars against broomrape from six populations

Three melon cultivars (KR1326, Huangpi 9818, K1076) were used to evaluate the resistance against broomrape from six populations (**Supplementary Table 2**) in pot experiments. The KR1326 and Huangpi 9818 cultivars were selected as potential resistant candidates showing postattachment resistance, and K1076 was selected as a susceptible cultivar based on the results of rhizotron, pot, and field experiments. A 1.5 L plastic pot was filled with 1.5 L of a mixture of compost, vermiculite, and sand (2:1:1 v:v:v) together with *P. aegyptiaca* seeds (50 mg seeds). Growth conditions and measurements of traits were the same as in the pot experiment.

### Collection of root exudates and extraction of root exudates

The collection method was as reported in Yoneyama et al. (2008) with several changes. Furthermore, 2-week-old seedlings (n = 12) of each cultivar (KR1326, K1076) were transferred to a 12-hole hydroponic box (16 × 20 × 7 cm, width × length × height (W × L × H)) containing 750 ml of 1/2 Hoagland nutrient solution as the culture medium in a growth chamber with a day/night regime of 16/8 h and a 10 000 Lx light source at 25°C. The Hoagland medium was exchanged every 3 d. After 10 d, melon seedlings were transferred to 1/2 Hoagland medium without P and grown for 3 d. Then, 3 d later, the roots of seedlings were rinsed with tap water, and tap water was used as the culture medium. Root exudates were collected every 3 d, four times in total, and each cultivar was repeated three times. Root exudates were immediately extracted with an equal volume of ethyl acetate three times. The ethyl acetate extracts were combined, dried over anhydrous MgSO_4_, and concentrated *in vacuo*. These crude extracts were stored in sealed glass vials at 4°C until use.

At the end of the final extraction, 2 g of the hydroponically cultivated melon roots were collected and used for quantitative detection of strigolactones (strigol and 5-deoxystrigol) in melon roots. After liquid nitrogen freezing and grinding, 15 ml of precooled acetone was added to the roots. The mixture was vibrated for 30 min by ultrasound, vortexed and mixed, and extracted overnight at 4°C in a shaker. After centrifugation (18514 g for 5 mins at 4°C), the acetone supernatant was taken and evaporated by nitrogen flow. When dry, 2 ml 20% acetone was added, and the solution was passed through an HLB (Hydrophile-Lipophile Balance) column (the passed fraction contained SLs) and repeated three times. Then, 2 ml of 100% acetone was added. Finally, the acetone was blown dry using nitrogen, re-dissolved with 200 μl of acetonitrile, and detected by HPLC-MS/MS (Yoneyama et al., 2008; Motonami et al., 2013). Characterization of strigolactones in the roots of melon was conducted by comparing retention times of germination stimulants on HPLC with those of synthetic standards (strigol and 5-deoxystrigol, strigolab) and by using HPLC-MS/MS.

### Germination assays

Germination assays of *P. aegyptiaca* (Plot 2) and *O. cumana* seeds were conducted as reported previously (Bao et al., 2017). The crude extract of root exudates was dissolved with 1 ml isopropanol and then diluted with sterile water 4, 10, 1 × 10^2^, 1 × 10^3^, 1 × 10^4^, 1 × 10^5^, and 1 × 10^6^ times. Then, 1 × 10^-7^ M GR24 (positive control) and sterile water (negative control) were used ascontrols. To exclude the effect of isopropanol on seed germination, isopropanol (4, 10, 1 × 10^2^, 1 × 10^3^, 1 × 10^4^, 1 × 10^5^, and 1 × 10^6^ times, diluted with sterile water) was also assessed. Then, 500 μl of the test solution was used for *P. aegyptiaca* germination, with three replications for each test solution. The germination rates of *P. aegyptiaca* and *O. cumana* seeds were examined with a stereo microscope at 7 d after treatment.

### Cross-sectioning and staining

The characterization of the resistant/susceptible phenotypes of melon was conducted by rhizotron infection experiments. The melon-*P. aegyptiaca* system was observed and photographed with a stereo microscope (Zeiss Discovery.V20) equipped with a digital camera (AxiaoCam ICc 5).

To examine the stage of parasite development on the host roots, small sections of tissue were dissected from the host root system at the S4 and S5 stages. The fresh root samples were fixed in FAA (5% formaldehyde + 10% acetic acid + 50% ethanol, in water) for 48 h. Paraffin sections were prepared as described previously (Bai et al., 2020). Alcian green-safranin double staining of sections was performed as described previously (Yoshida and Shirasu, 2009).

The sections were examined under a light microscope (Axio Imager M2, Zeiss, Germany) and photographed using a XiaoCam MRC digital camera. For visualization of total lignin autofluorescence, the sections were observed by epi-fluorescence under excitation at 450–490 nm. To obtain comparable images, fluorescence intensity was kept constant between samples.

For callose detection, root segments with attached haustoria were excised, and we hand-sectioned plants at 200–300 μm thickness using razorblades and kept the sections in 4°C water before staining. The sections were stained for 15–30 min in a solution of 0.1% aniline blue fluorochrome in water (Bordallo et al., 2002). Aniline blue fluorochrome was used for callose detection under UV fluorescence (340–380 nm).

### Statistical analysis

ANOVA was performed using the SPSS 19.0 software. In the rhizotron evaluation and pot experiments, the number of *P. aegyptiaca* seedlings in each growth stage was examined by Tukey’s HSD test with melon cultivar as a factor. The data of rhizotron evaluation were presented as relative means ± SE in the form of graphs using the statistical package R (version 4.1.2, ggplot2). In the pot experiments, PH, SD, GDW, GFW, and RDW of melon cultivars were analyzed by Tukey’s HSD test. Student *t* test *P* values > 0.10, < 0.1, < 0.05 and < 0.01, < 0.001 were marked by NS, +, *, **, and ***, respectively. Photoshop (version 2019) was used for image processing. The details concerning statistics used in data analyses performed in this study are described in the respective sections of results and methods.

## Results

### Evaluation of melon resistance to *P. aegyptiaca* in rhizotron assays

**Figure 1:**
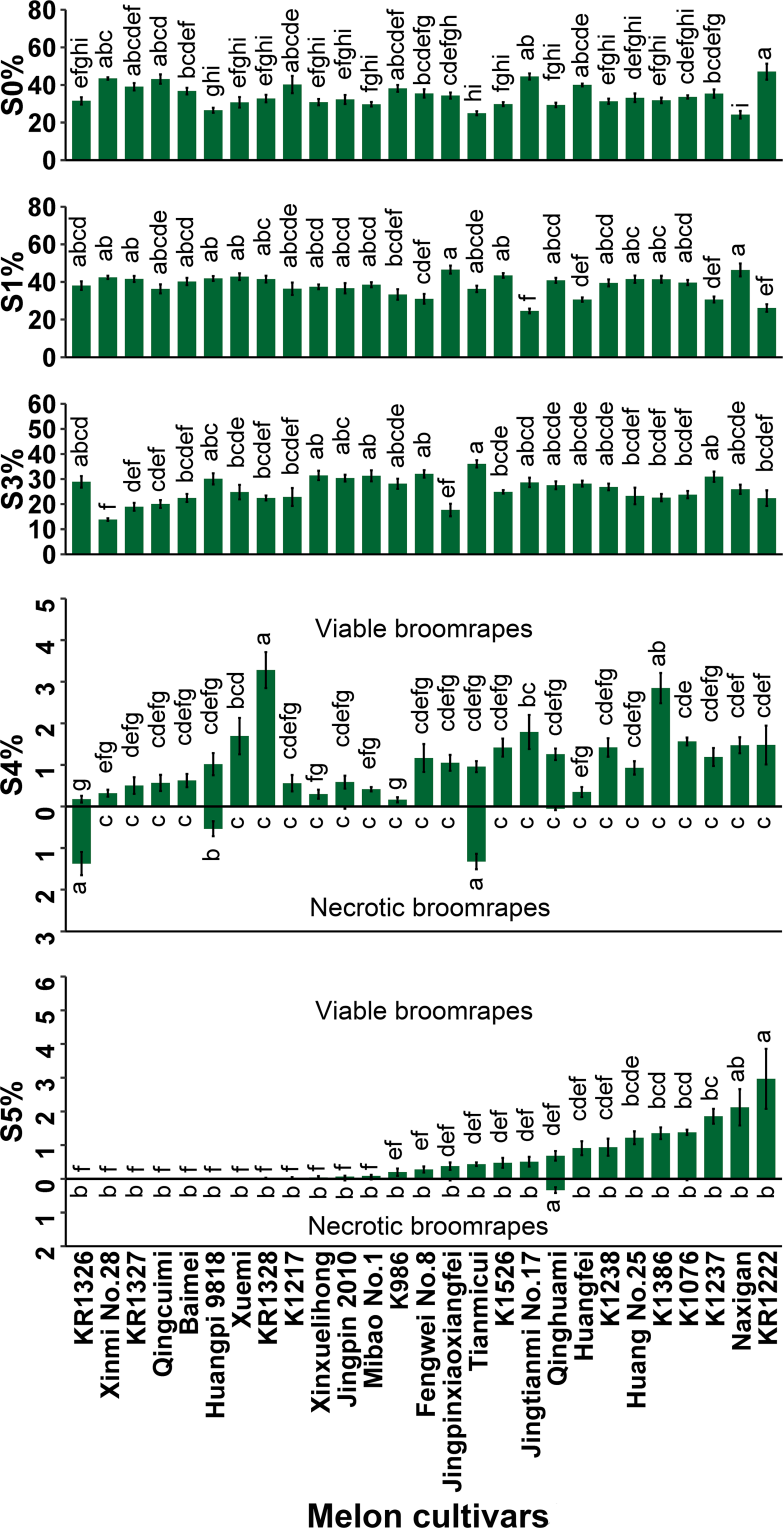
Percentages of *P. aegyptiaca* at different growth stages on roots of 27 melon cultivars at 35 DAI. S0, *P. aegyptiaca* seeds were not germinated; S1, seeds germinated but failed to attach to the melon roots; S3, seedlings had firmly adhered to or penetrated the host roots but had not formed a vascular connection; S4, tubercle stage, the parasite had formed a vascular connection to the host roots; S5, the “spider” stage (tubercles with secondary roots) ≤ 1cm. Vertical bars represent the mean ± SE, while letters represent mean separation at *P* ≤ 0.05 by Tukey’s HSD test in each growth stage.

Large phenotypic differences were observed among 27 melon cultivars in the various steps of development, from *P. aegyptiaca* seed germination to establishment on melon roots (**Figure 1**). Of the seed inocula, failure to germinate was highest in KR1222 (47.05%) and lowest in Naxigan (24.15%).

Development of the parasite at stage S1 (seeds germinated but fail to attach to host roots) was common among all cultivars. Arrested development at this stage was highest in Jingpinxiaoxiangfei (46.54%) followed by Naxigan (46.35%) and lowest in Jingtianmi No.17 (24.55%). Many of the attachments did not develop past the S1 stage due to a lack of haustorium formation.

Arrested development of the parasite at stage S3 was observed, where seeds had firmly adhered to or penetrated the host roots but had not formed a vascular connection. Successful attachment but without the formation of a tubercle was highest in Tianmicui (36.08%) and lowest in Xinmi No.28 (13.84%).

The arrested development of *P. aegyptiaca* at stage S4, necrosis of the tubercles, was observed in five cultivars (Jingpin 2010 0.027%, Qinghuami 0.052%, KR1326 1.37%, Huangpi 9818 0.54%, Tianmicui 1.32%) and was highest in KR1326 (1.37%) (**Figure 1**; **Supplementary Figure 3F**). In this cultivar, 1.55% of the inoculated parasites underwent arrested development at stage S4, of which the necrotic tubercles of broomrapes accounted for 88.39%.

The *P. aegyptiaca* seedlings at stage S5, the “spider” stage (tubercles with secondary roots), were observed in 20 cultivars and varied widely among cultivars, representing 0–2.97% of the original inocula (**Figure 1**). In seven cultivars (Xuemi, Huangpi 9818, Baimei, Qingcuimi, KR1327, Xinmi No.28, and KR1326), the inoculated parasites could not grow up to the S5 stage. Some necrotic broomrape at the S5 stage was observed in five cultivars (K1076, Qinghuami, Jingpinxiaoxiangfei, Mibao No.1, and Jingpin 2010).

### Evaluation of melon resistance to *P. aegyptiaca* in pot assays

**Figure 2:**
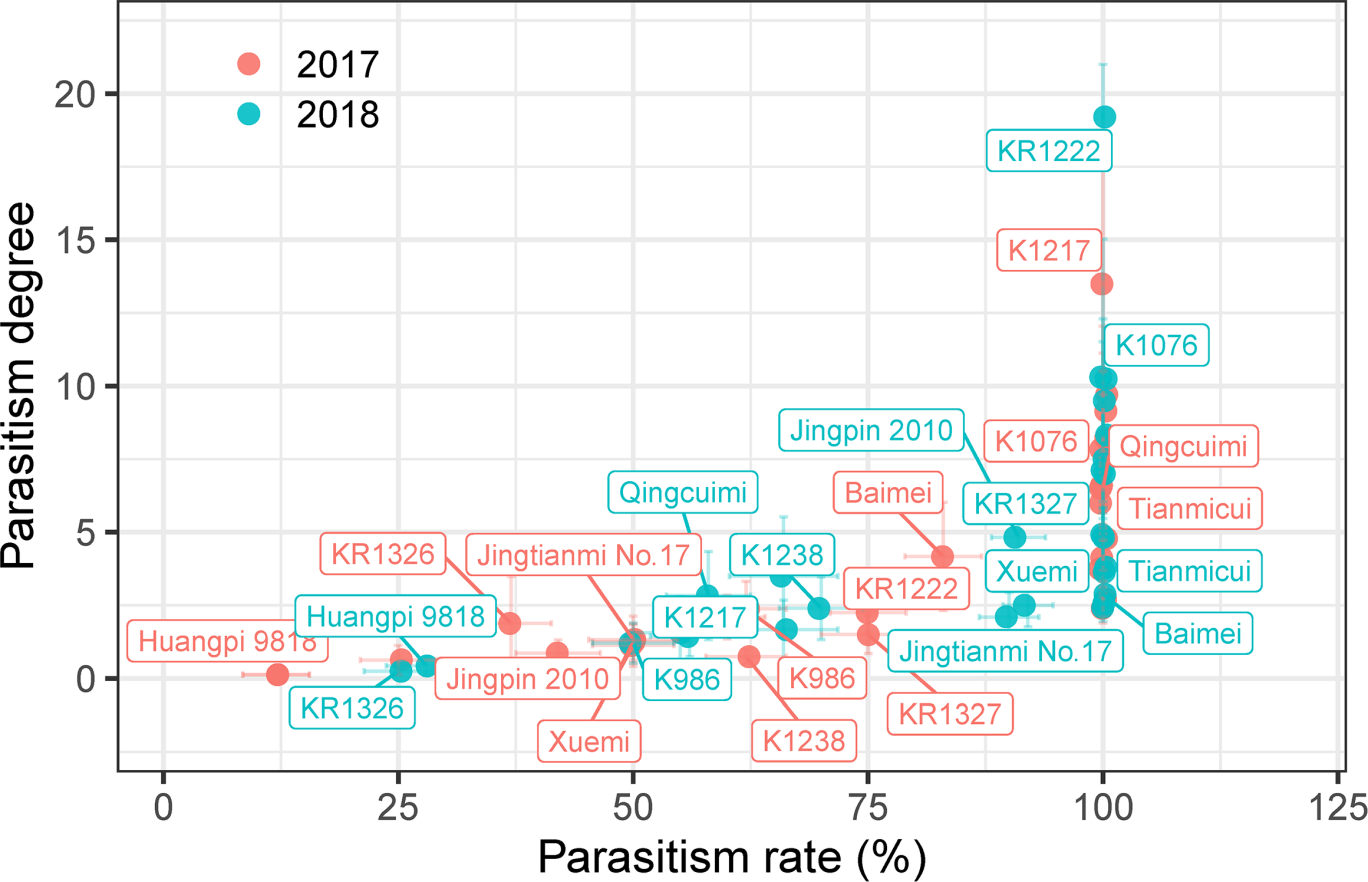
Parasitism degree and parasitism rate of the different melon cultivars in pot experiments. Vertical and horizontal bars indicate standard errors of parasitism degree and rate, respectively (*n* = ~3–8, biological replicates). Only some representative melon cultivars are shown in the figure.

The number and biomass of *P. aegyptiaca* and the plant height, stem diameter, and dry weight parameters of the host were investigated to evaluate the resistance of melon to *P. aegyptiaca* in pot experiments (**Figure 2** and **Supplementary Tables 5**, **6**).

As shown in **Figure 2**, the results of the 2018 pot experiment were similar to those in 2017. Among these cultivars, the parasitism rate and degree of nine cultivars (K1217, KR1328, Mibao No.1, Naxigan, Qingcuimi, Tianmicui, Baimei, Fengwei No.8, and Xinmi 28) in 2017 pot experiments were higher than those in 2018, and the parasitism rate and degree of the other cultivars were lower than those in 2018. The mean values of parasitism rate and parasitism degree in the two years varied from 20.54 ± 10.69 to 100.00 ± 0.00 and from 0.27 ± 0.15 to 10.17 ± 2.42, respectively. According to the mean values of parasitism rate and parasitism degree of 27 melon cultivars, these could be divided into four groups: 1) a highly sensitive group comprising KR1222 with the highest parasitism degree (10.17 ± 2.42); 2) a sensitive group of 16 cultivars including K1076, K1217, Huang No.25, K1237, KR1328, Huangfei, Tianmicui, and others; 3) a resistant group composed of K1386, K1526, Jingpin 2010, Xinxuelihong, K986, Xuemi, Jingtianmi No.17, and K1238; 4) a highly resistant group including KR1326, and Huangpi 9818. Huangpi 9818 showed a lower parasitism rate and degree over the two consecutive years, significantly lower than other cultivars, and had no significant difference with KR1326.

As shown in **Supplementary Table 5**, *P. aegyptiaca* infection in various melon hosts was variable with respect to the number and biomass of *P. aegyptiaca* attachments. Susceptible melon had more broomrape at S6 and S7 stages and greater broomrape biomass. The arrested development of broomrape at S4, the tubercle stage, was observed in Xuemi and KR1326 and was lowest in KR1326 (0.50 ± 0.27). There were nine cultivars (K1526, Jingpin 2010, KR1328, Huangpi 9818, Mibao No.1, Qinghuami, Baimei, Jingtianmi No.17, and K1217) in which arrested development of *P. aegyptiaca* was observed at S5, the “spider” stage (tubercles with secondary roots). The arrested development of the parasite at S6 (sprout stage) was observed in 11 cultivars (K1237, K1076, KR1222, Jingpinxiaoxiangfei, Tianmicui, Xinmi 28, Fengwei No.8, K986, KR1327, and Qingcuimi, K1238). In five cultivars (Huang No.25, Huangfei, Naxigan, Xinxuelihong, and K1386), broomrape developed to the S7 stage (spike stage). The lowest number of broomrape attachments (0.50 ± 0.27) and broomrape biomass were observed in KR1326. The heaviest attachment dry weight of broomrape was observed in Huang No. 25 and was significantly higher than the other cultivars, followed by K1237, Huangfei, K1076, Naxigan, and KR1222. The attachment dry weight of broomrape on K1238, Huangpi 9818, Mibao No.1, Qinghuami, Baimei, Xuemi, Jingtianmi No.17, K1217, and KR1326 was close to zero.

The broomrape had no significant adverse effects (*P* > 0.10) on the total aboveground parts dry weight (GDW), root dry weight (RDW), plant height (PH), or stem diameter (SD) in Qingcuimi, Jingpin 2010, Tianmicui, KR1327, KR1326, Xuemi, Huangpi 9818, and Baimei (**Supplementary Table 6**). However, there were significant differences (*P* < 0.001, 0.01, or 0.05) in GDW, RDW, and PH in KR1222, K986, K1076, Huangfei, K1237, Naxigan, Jingpinxiaoxiangfei, and Huang No.25. The broomrape also had no significant adverse effects (*P* > 0.10) on the GDW in Xinxuelihong, Fengwei No.8, Xinmi 28, K1526, Qinghuami, Mibao No.1, K1238, K1217, KR1328, Jingtianmi No.17, Qingcuimi. The parasite reduced the RDW of Xinxuelihong, Fengwei No.8, Xinmi 28, K1526, Qinghuami, and Mibao No.1 by 45.71% (*P* < 0.01), 54.17% (*P* < 0.01), 44.83% (*P* < 0.05), 43.75% (*P* < 0.05), 28.57% (*P* < 0.05), and 36.36% (*P* < 0.05), respectively. *P. aegyptiaca*, also significantly reduced PH of eight cultivars, and the observed reductions were 17.65% (*P* < 0.01), 9.66% (*P* < 0.05), 41.50% (*P* < 0.01), 26.03% (*P* < 0.05), 21.42% (*P* < 0.001), 44.54% (*P* < 0.01), 16.55% (*P* < 0.05), 28.72% (*P* < 0.05) for Xinxuelihong, Fengwei No.8, Xinmi 28, K1526, K1238, K1217, KR1328, and Jingtianmi No.17, respectively. In addition, SD was reduced by 27.04% (*P* < 0.01) and 10.68% (*P* < 0.05) in Naxigan and Huang No.25, respectively.

Taken together, our results suggested that for the cultivars that were screened, K1217, Jingpin 2010, KR1326, Xuemi, Huangpi 9818, Baimei, and Jingtianmi No.17 were promising cultivars against *P. aegyptiaca* with low parasitism rate and parasitism degree and the growth is less affected by broomrape. In particular, Huangpi 9818 and KR1326 could provide valuable resistance against *P. aegyptiaca*.

### Field-site screening of melon cultivars for *P. aegyptiaca* Resistance

**Figure 3:**
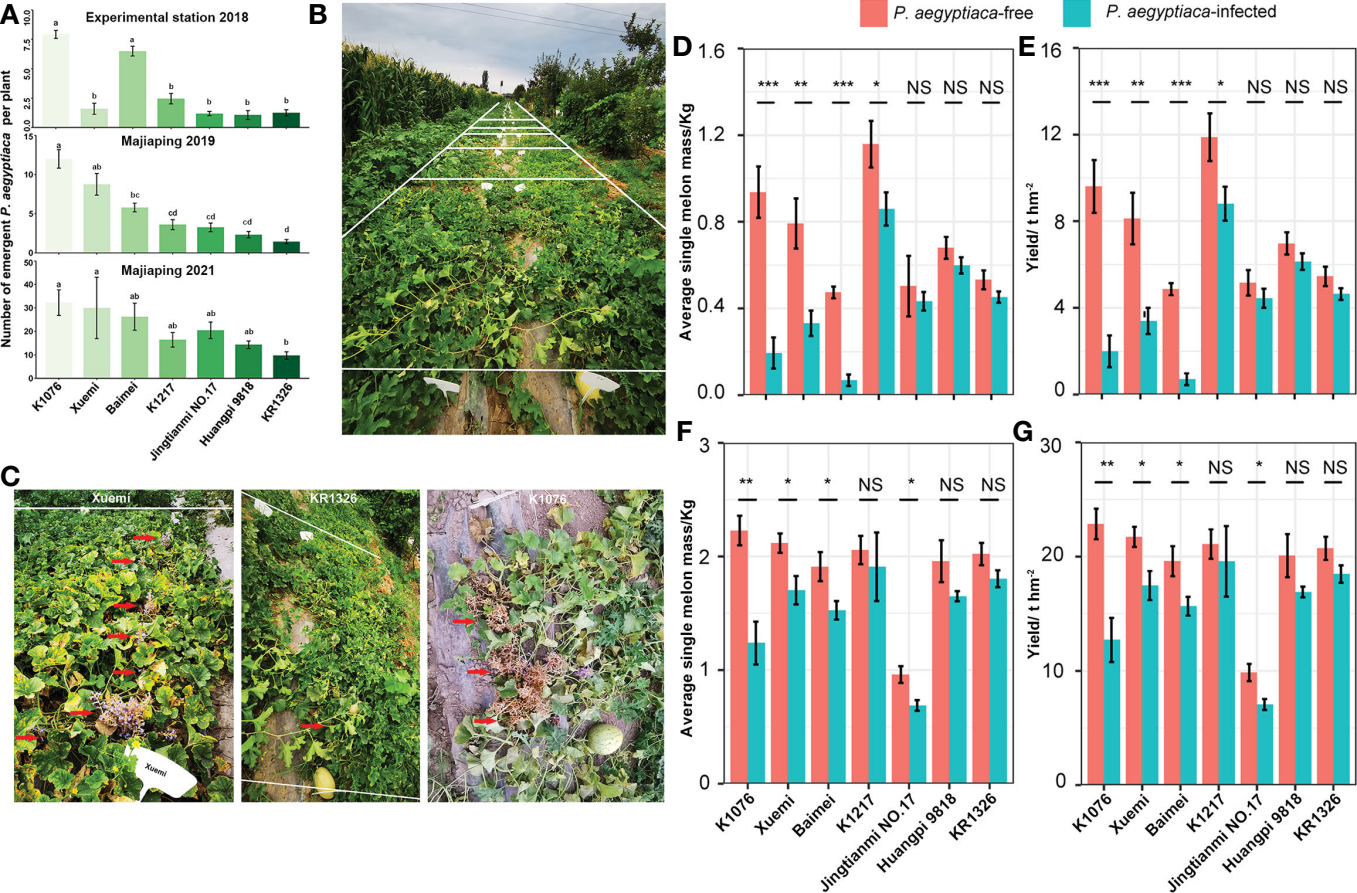
The resistance response of melon cultivars to *P. aegyptiaca* at the experimental station and Majiaping field sites. K1076, Xuemi, Baimei, K1217, Jingtianmi No.17, Huangpi 9818, and KR1326 were used in the field trials. **(A)** The number of emerged *P. aegyptiaca* plants per cultivar for the experimental station in 2018 and Majiaping in 2019 and 2021. Data presented are means ± SE. The letters above each bar indicate significantly different (*P* ≤ 0.05) groups after Tukey’s HSD test. **(B)** Contrasting *P. aegyptiaca* infection levels in the *P. aegyptiaca* screening trial at Majiaping (July 2021) replicate 3; sub-plots, representing cultivars are delimited by white lines. **(C)** The growth of Xuemi, KR1326, and K1076 at Majiaping (July 2021). Red arrows represent the emerged *P. aegyptiaca* shoots. **(D, F)** Single melon mass (Kg) per cultivar for the experimental station in 2018 and Majiaping in 2021. **(E, G)** Melon yield (t ha^-1^) per cultivar for the experimental station in 2018 and Majiaping in 2021. Data presented are means ± SE. NS, *, **, *** indicate significant differences at *P* > 0.05, *P* < 0.05, *P* < 0.01, and *P* < 0.001 respectively, by Student’s *t* test within a variety.

Based on the numbers of aboveground *P. aegyptiaca* observed in the field, KR1326 was the most resistant cultivar in both the experimental station (2018) and Majiaping (2019, 2021) (**Figure 3A**). In 2018, the numbers of aboveground *P. aegyptiaca* of KR1326, Huangpi 9818, Jingtianmi No.17, K1217, and Xuemi (1.25 ± 0.26, 1.06 ± 0.38, 1.18 ± 0.18, 2.45 ± 0.44, 1.59 ± 0.47) were significantly lower than those of K1076 and Baimei.

In 2019, the aboveground numbers of *P. aegyptiaca* of KR1326 (1.44 ± 0.26) were notably lower than those of K1076, Xuemi, and Baimei (12.00 ± 1.18, 8.75 ± 1.39, and 5.80 ± 0.56) but not significantly different from Huangpi 9818, K1217, and Jingtianmi No.17 (2.33 ± 0.40, 3.61 ± 0.64, and 17 3.26 ± 0.56).

In 2021 (**Figures 3A, B**), the numbers of aboveground *P. aegyptiaca* of all cultivars were significantly higher than in 2019. The lowest was KR1326 (9.74 ± 1.58) followed by Huangpi 9818 (14.32 ± 1.61) and K1217 (16.43 ± 3.12), and the highest was K1076 (32.24 ± 5.46). The resistance to *P. aegyptiaca* among cultivars varied; for example, Xuemi, KR1326, and K1076 showed different resistance in 2021 (**Figure 3C**). KR1326 exhibited the highest resistance to *P. aegyptiaca* and was more resistant than Jingtianmi No.17 and Xuemi (**Figures 3A, C**). This cultivar had very few emerging broomrape shoots through the soil (**Figure 3C**).

In summary, the field experiments confirmed the laboratory results, i.e., that some melon cultivars were resistant to *P. aegyptiaca.* In particular, KR1326 showed the most resistance at all field sites.

### Effects of *P. aegyptiaca* infestation on melon yield

In the *P. aegyptiaca* infested fields, the average single melon weight and yield per cultivar ranged from 0.07 to 0.86 kg (average: 0.42 kg) and 0.70 to 8.81 t ha^-1^ (4.3 t ha^-1^) in 2018, and from 0.69 to 1.91 kg (average: 1.55 kg) and 7.06 to 19.58 t ha^-1^ (15.40 t ha^-1^) in 2021 (**Figures 3D–G**). The single melon weight and yield per cultivar in 2018 were significantly lower than in 2021. This difference was due to planting density and soil fertility.

Broomrape affected the yields of all melon cultivars compared with their controls, and the effect was more severe on the susceptible cultivars. Single melon weight and yield were significantly decreased for K1076, Xuemi, and Baimei in 2018 (at the experimental station) and 2021 (at Majiaping). Huangpi 9818 and KR1326 yield decreased by 12.03% and 14.98% in 2018 and by 15.88% and 10.88% in 2021, respectively. However, in some cultivars, the effects of broomrape were more significant and had a close relationship with the field trial results. For example, broomrape infestation reduced the K1217 yield by 25.87% at the experimental station in 2018, but not at Majiaping in 2021. Jingtianmi No.17 yield decreased by 13.94% (2018) with nonsignificant differences compared with the control but decreased significantly by 28.41% in 2021. These results suggest that broomrape altered the yields of susceptible cultivars more drastically compared to resistant cultivars. In addition, Huangpi 9818 and KR1326 yield loss was minor in K1076 in all field sites.

### Resistance response of different melon cultivars against six broomrape populations

The results of rhizotron and pot experiments showed that KR1326 and Huangpi 9818 had better resistance to broomrape from Plot 2 that had been collected from processing tomatoes growing in 3rd company, Junhu, Xinjiang. To determine whether the resistance of KR1326 and Huangpi 9818 was broad-spectrum or specific to the Plot 2 *P. aegyptica* population, KR1326, Huangpi 9818, and K1076 (the most susceptible cultivars) were infected with *P. aegyptiaca* from six different populations (including Plot 2).

**Figure 4:**
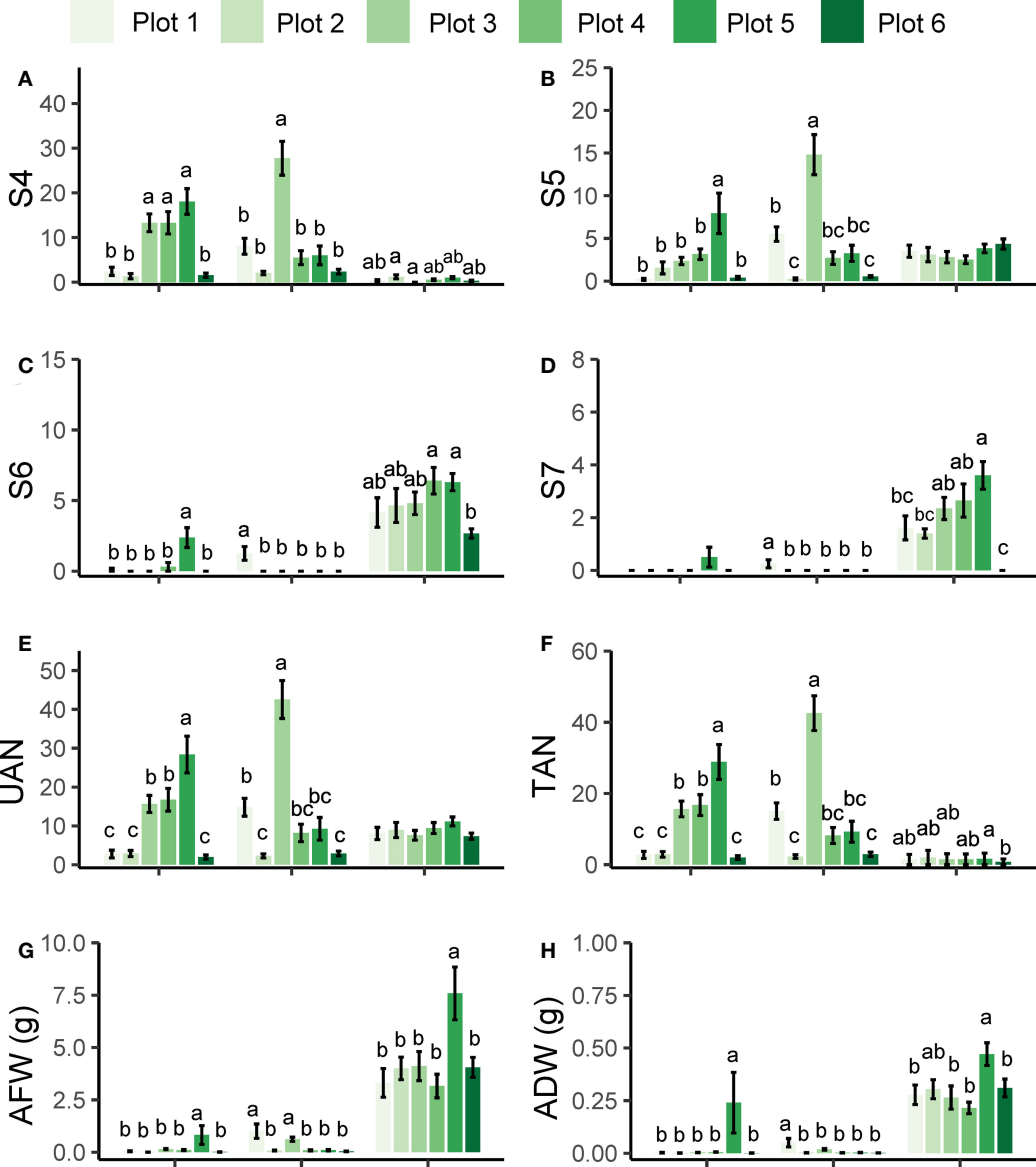
Development and biomass of six *P. aegyptiaca* populations attached to the roots of KR1326, Huangpi9818, and K1076 at 60 DAI. **(A–D)** The number of *P. aegyptiaca* at S4, S5, S6, S7 stages per host plant. **(E)** Total number of underground attachments per host plant (UAN). **(F)** Total number of *P. aegyptiaca* attachments per plant (TAN). **(G)** Fresh weight of total attachments per host plant. **(H)** Dry weight of total attachments per host plant. Vertical bars represent the mean ± SE, while letters represent mean separations at *P* ≤ 0.05 by Tukey’s HSD test (*n* = 10 biological replicates).

**Figure 4** shows the attachment number and the biomass of each *P. aegyptiaca* group on the different melon cultivars. KR1326 and Huangpi 9818 showed the same pattern of resistance to Plot 1, Plot 2, Plot 4, and Plot 6 broomrape populations, although they were collected from different host species and different regions (**Supplementary Table 2**). K1076 was very susceptible to all populations of *P. aegyptiaca*, especially to the Plot 5 population. The amount of *P. aegyptiaca* biomass (AFW and ADW) of Plot 5 on K1076 was significantly higher than for other broomrape populations. Similar to K1076, the number of Plot 5 *P. aegyptiaca* at S5 and S6 stages and the amount of *P. aegyptiaca* biomass on KR1326 were significantly higher than in plants treated with *P. aegyptiaca* from other populations. In addition, the pattern of resistance of Huangpi 9818 to Plot 3 *P. aegyptiaca* differed from those of Plot 1, Plot 2, Plot 4, Plot 5, and Plot 6 *P. aegyptiaca*. A large number of attachments (S4 and S5 stage) of broomrape were observed on Huangpi 9818 roots, significantly higher than in other broomrape populations. A small number of broomrape plants from Plot 1 developed to S6 and S7 stages, while the development of others stagnated at the S4 and S5 stages. These results suggested that the differences in the number of different developmental stages and biomass of parasites from the same *P. aegyptiaca* population on different hosts were related to the strength of host-*P. aegyptiaca* interactions. In addition, six *P. aegyptiaca* populations showed consistency in the resistance response of KR1326 and Huangpi 9818 to *P. aegyptiaca*.

### Impact of six *P. aegyptiaca* populations on the growth of susceptible and resistant melon cultivars

**Figure 5:**
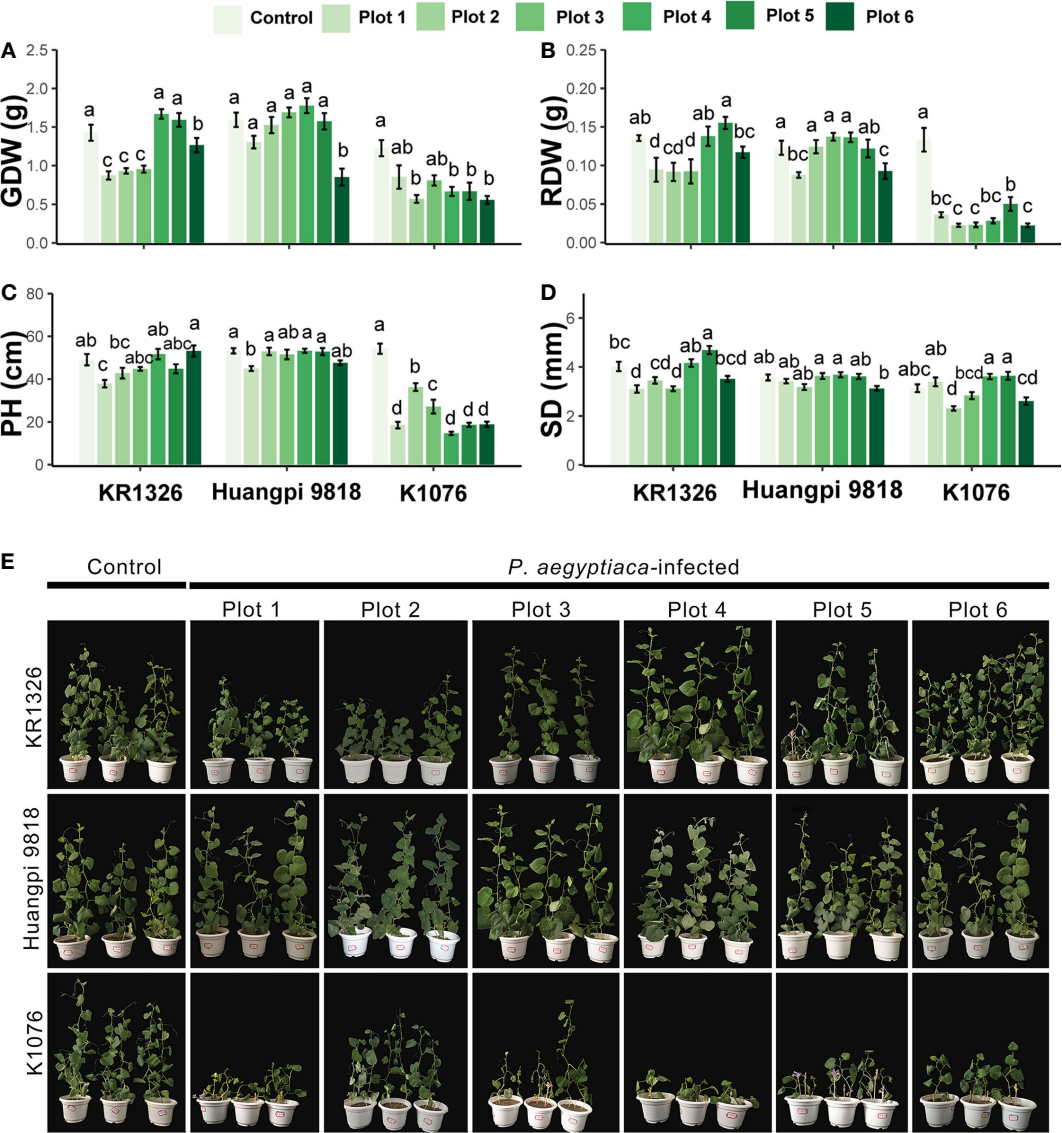
Effects of six *P. aegyptiaca* populations on the growth of different resistant melon cultivars. **(A)** Effects of *P. aegyptiaca* on melon total aboveground parts dry weight (GDW). **(B)** Effects of *P. aegyptiaca* on melon root dry weight (RDW). **(C)** Effects of *P. aegyptiaca* on melon plant height (PH). **(D)** Effects of *P. aegyptiaca* on melon stem diameter (SD). **(E)** Growth of melon after inoculation with or without broomrape from six populations. Vertical bars represent the mean ± SE, while letters represent mean separations at *P* ≤ 0.05 by Tukey’s HSD test (*n* = 10 biological replicates).

The plant height, stem diameter, and dry biomass of the infected melon cultivars were compared with those of the noninfected plants to evaluate the impact of *P. aegyptiaca* infection on the growth of the host. *P. aegyptiaca* affected the growth of KR1326, Huangpi 9818, and K1076, and more severely affected the susceptible cultivar K1076 (**Figure 5E**). The parasitism by broomrape had a greater effect on the total aboveground parts dry weight, root dry weight, and plant height of the hosts, while the effect on the stem diameter was less, although the difference was significant (**Figures 5A–D**). Among these three cultivars, the plant height, total aboveground parts dry weight, and root dry weight of the susceptible cultivar K1076 were most influenced by broomrape, being reduced by 33.17–72.97%, 30.51–54.63%, and 62.42–83.35%, respectively. In the two most resistant cultivars, KR1326 and Huangpi 9818, total aboveground parts dry weight, root dry weight, and plant height were slightly suppressed, but still significantly (**Figures 5A–D**). These results suggest that *P. aegyptiaca* altered the biomass of susceptible cultivars more compared to resistant cultivars.

### Evaluation of the germination-inducing activity of preattachment resistance

**Figure 6:**
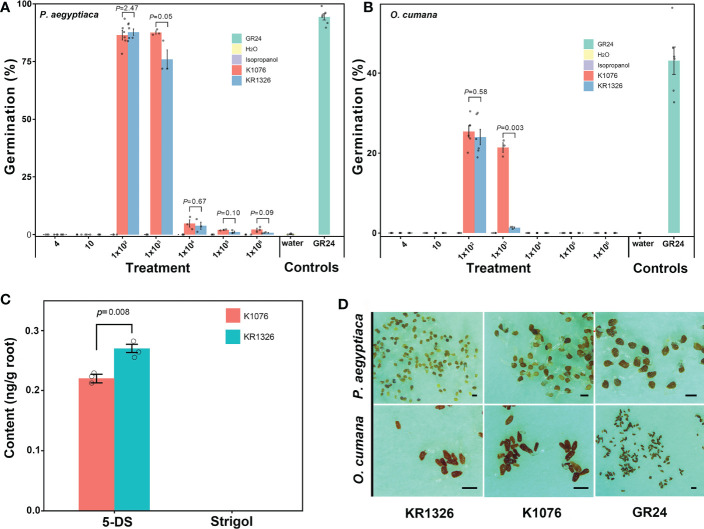
Differences in root exudate of susceptible (K1076) and resistant (KR1326) melon cultivars for germination induction of *P. aegyptiaca* **(A)** and *O. cumana* **(B)** seeds and differences in contents of 5-DS and Strigol in roots **(C)**. The crude extract of K1076 and KR1326 root exudates was dissolved with 1 ml isopropanol and then diluted with sterile water 4, 10, 1 × 10^2^, 1 × 10^3^, 1 × 10^4^, 1 × 10^5^, and 1 × 10^6^ times (n ≥ 3 replicates). Seeds were treated with isopropanol diluted with sterile water at 4, 10, 1 × 10^2^, 1 × 10^3^, 1 × 10^4^, 1 × 10^5^, and 1 × 10^6^ times as negative controls. Seeds treated with or without GR24 at 1 × 10^-7^ M were always included as positive and negative controls, respectively. **(C)** Amounts of 5-DS and Strigol (ng/g) in roots (2g) of K1076 and KR1326 obtained in hydroponics (n=3 biological replicates). **(D)** 1 × 10^2^-time concentrated root exudates from K1076 and KR1326 and GR24 induced germination of *P. aegyptiaca* and *O. cumana.* Experiments were repeated at least three times. Scale bar, 500 μm. Vertical bars represent the mean ± SE and *P* value was conducted by Student *t* test.

The resistant cultivar KR1326 was selected for detailed germination-inducing activity and microscopic characterization. The germination induction effect of KR1326 root exudates in comparison with K1076 on seeds of *P. aegyptiaca* and *O. cumana* is shown in **Figure 6**. In all cases, null germination was observed when both *P. aegyptiaca* and *O. cumana* seeds were treated with distilled water (negative control). After treatment with GR24 at 1 × 10^-7^ M (positive control), the two parasites’ seed germination rates were 94.29% ± 1.35% and 42.79% ± 1.51%, respectively.

The results showed that different concentrations of isopropanol did not affect the germination of *P. aegyptiaca* or *O. cumana* seeds (**Figures 6A, B**). At the same time, the data showed that the germination percentage of *P. aegyptiaca* was higher than that of *O. cumana* treated with K1076 and KR1326 root exudates. Treatments with 4, 10, 1 × 10^4^, 1 × 10^5^, and 1 × 10^6^ times the concentration of root exudates of K1076 and KR1326 induced negligible to little (0–4.8%) germination, while 1 × 10^2^ times the concentration of root exudates of K1076 and KR1326 induced high germination of *P. aegyptiaca* seeds (86.42 ± 2.10% and 87.70% ± 1.37%, *P =* 2.47) and *O. cumana* seeds (24.39% ± 4.46% and 21.34.01% ± 3.67%, *P =* 0.58) (**Figures 6A, B, D**). In addition, 1 × 10^3^ times the concentration of root exudates from K1076 (87.53% ± 0.70%) also highly induced seed germination of *P. aegyptiaca*, and the percentage was higher than in KR1326 (75.93% ± 4.05%) (*P =* 0.05). However, germination of *O. cumana* seeds with 1 × 10^3^ times the concentration of root exudates from K1076 (18.05% ± 2.58%) was significantly higher than that induced by KR1326 (1.33% ± 0.17%) (*P =* 0.003).

HPLC-MS/MS analyses of 5-DS and strigol of root extracts of K1076 and KR1326 are shown in **Figure 6C**. The 5-DS was detected in roots of both K1076 and KR1326. The concentration in KR1326 root extracts was 0.27 ng/g, slightly higher than for K1076 (0.22 ng/g) (*P =* 0.008). Strigol was not detected in the roots of either melon cultivar.

### Histological analyses

**Figure 7:**
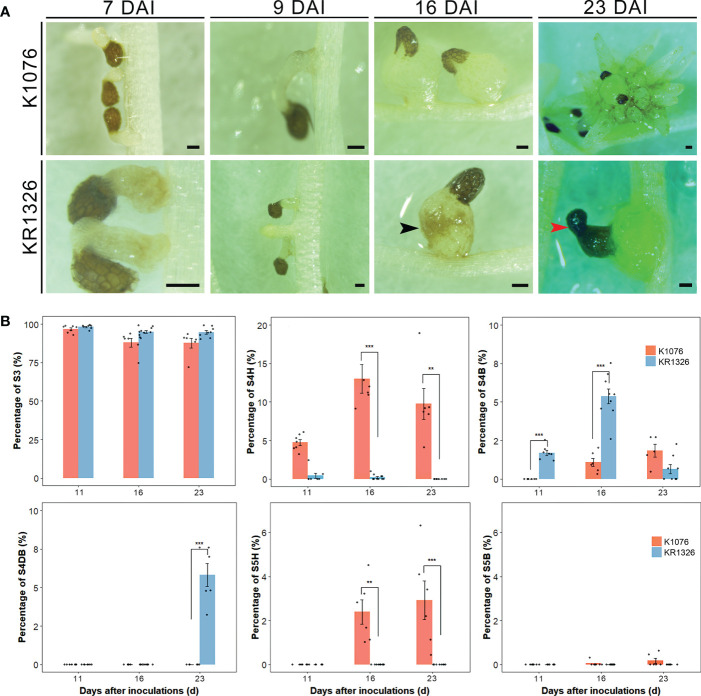
Differential response of K1076 and KR1326 to parasitism by *P. aegyptiaca*. **(A)** Representative photographs illustrating the phenotypic response of K1076, a susceptible cultivar, and KR1326 a resistant cultivar, to parasitism by *P. aegyptiaca.* Shown are the appearance of broomrape and the attachment sites at 7, 9, 16, and 23 DAI (the broomrape was in S2, S3, S4, and S5 stages, respectively). Bars = 200 μm. **(B)** Measured percentage of each different phenotypic event category during the interaction of *P. aegyptiaca* with resistant (KR1326) and susceptible (K1076) melon roots at 11, 16, and 23 DAI (n = 5~10). The abbreviation of the phenotypic event categories are as follows: S3, seeds had firmly adhered to or penetrated the host roots but had not formed a vascular connection; S4H, healthy tubercles; S4B, tubercle slight discoloration (black arrow); S4DB, tubercle severely browning or necrosis (red arrow); S5H, broomrape is healthy in the “spider” stage; S5DB, broomrape shows severe browning or necrosis in the “spider” stage. Data presented are means ± SE. *, **, *** indicate significant differences at *P* < 0.05, *P* < 0.01, and *P* < 0.001 respectively, by Student’s *t* test within a variety.

Melon cultivars displayed striking differences in their ability to support the growth and development of *P. aegyptiaca*. To understand how melon cultivars resisted *P. aegyptiaca* parasitism, we performed microscopic observations of *P. aegyptiaca* haustoria on K1076 and KR1326 roots at 7, 9, 16, and 23 DAI. Here, 7- and 9-d-old parasites on KR1326 and K1076 were phenotypically indistinguishable (**Figure 7A**). No clear discoloration was found on the roots of either cultivar at the parasite attachment site at this early stage (**Figure 7A**). This indicates that the hypersensitive response (HR) was not involved in the resistance response. At 16 DAI, broomrape had successfully penetrated the K1076 and KR1326 root cortex and established connections with the host vascular system. There was the formation of an enlarged tubercle, an indication of the successful vascular connection between the host and parasite. Compared with K1076, broomrape on KR1326 only showed minor distinguishable phenotypic differences of slight discoloration or browning on KR1326 (**Figure 7A**, black arrow). However, resistant interactions had significantly more discolored tubercles (S4B) (*P* < 0.001) and lesser healthy tubercles (S4H) (*P* < 0.001) by comparison to susceptible interactions (**Figure 7B**). However, at 23 DAI, dramatically different phenotypic responses were observed among the susceptible and resistant interactions (*P* < 0.01 for S4H; *P* < 0.001 for S4DB and S5H). The parasite’s secondary roots thrived in susceptible interactions, and the haustoria significantly expanded (**Figure 7A**). In stark contrast, the appearance of the parasite on KR1326 showed severe browning and was significantly smaller (**Figure 7A**, red arrow) than the parasite on K1076. This indicates that the resistance of KR1326 against *P. aegyptiaca* may be related to the inhibition of the growth and development of the parasite.

**Figure 8:**
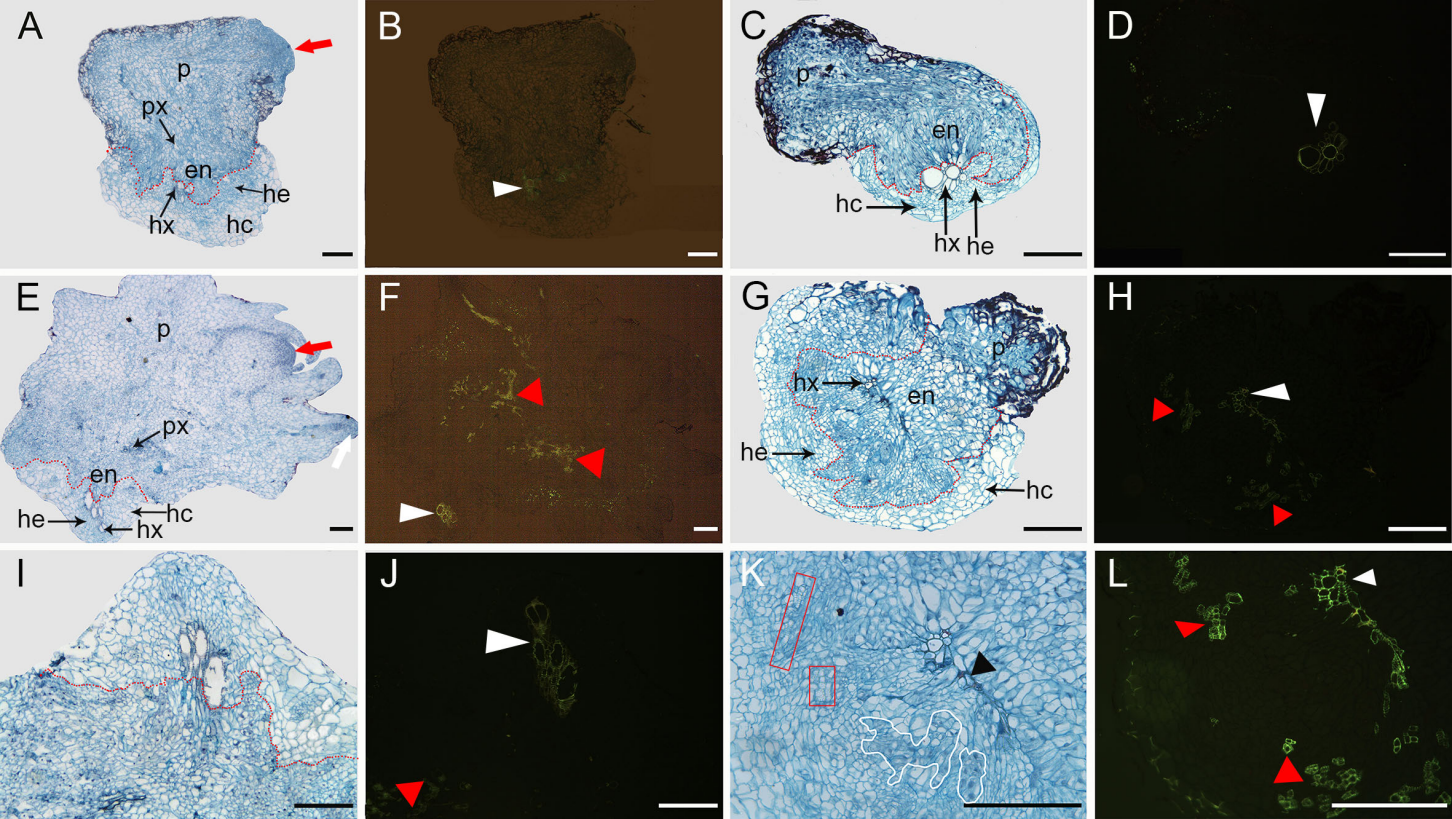
Transverse sections through the root and *P. aegyptiaca* attachment in compatible and incompatible interactions at 16 and 23 DAI. **(A, E)** Light micrograph of a cross-section in a healthy broomrape on K1076 (16 and 23 DAI). **(I)** Detail of **(E)**. **(B, F, J)** The same as **(A, E, I)** under fluorescence microscopy (450–490 nm). **(C, G)** Light micrograph of a cross-section in a slightly discolored and severely browned tubercle on KR1326 (16 and 23 DAI). **(K)** Detail of **(G)**. **(D, H, L)** The same as **(C, G, K)** under fluorescence microscopy (450–490 nm). p, parasite (*P. aegyptiaca*); px, parasite xylem; hx, host xylem; he, host endodermis; hc, host root cortex; en, endophyte. The red arrow indicates the young stem meristem of the broomrape; the white arrow indicates the secondary root meristem; the white triangle indicates autofluorescence of the host xylem; the red triangle indicates autofluorescence of the xylem of the broomrape; the black triangle indicates the accumulation of secondary metabolites in the host cell wall; the red box indicates parasite xylem, and a white border indicates parenchyma cells of the parasite. Bars = 200 μm.

To clarify the changes in cell structure associated with the resistance mechanism by which the host root inhibits parasite development, histological analyses were performed on sections of infected roots containing an attached parasite tubercle (**Figure 8**). At 16 DAI, in K1076 and KR1326 roots the *P. aegyptiaca* endophyte (en) and xylem cells invaded the central cylinder and established vascular connections (**Figures 8A, C**). The cells of broomrape occupied the host central cylinder tissues. Compared to the resistant cultivar KR1326, a large number of densely blue-stained cells (red arrow) are present in the apical part of the parasite in K1076 roots, indicating that the haustorial tissues had begun to differentiate (**Figure 8A**). A transverse section through a haustorium on K1076 at 23 DAI showed well-developed xylem (px), young stem meristem (red arrow), and root meristem (white arrow) (**Figure 8E**). Ultraviolet (UV) autofluorescence further confirmed that lignin autofluorescence was more robust in the xylem tissues of parasites (red triangle) in K1076 roots (**Figure 8F**) than in KR1326 (**Figure 8H**).

In contrast, for the roots of KR1326, the haustorium differentiation of the parasite was not apparent. A more detailed observation of the resistant interactions showed that the host xylem cells in contact with the parasite intrusive cells presented a thickening of their walls stained intensely red with safranin (**Figure 8K**). We also observed the reddish coloration in the intercellular spaces between the host xylem and intrusive parasite cells (black triangles). This phenomenon may be caused by the accumulation of large amounts of secondary metabolites at this site. In addition, columns of discontinuous xylem (red box) and blue-stained parenchyma cells (white border) of the broomrape embedded between the endodermis and xylem of the host root were observed (**Figure 8K**). Observation of these sections under a fluorescence microscope showed an intense green fluorescence in both the xylem of the parasite inside the host (red triangle) and host (white triangle) (**Figure 8L**). The above results show that vascular continuity was established between parasite and host but that there was poor haustorium tissue differentiation on KR1326 compared with K1076. This raises the possibility that KR1326 either lacks key specific primary/secondary metabolites/signals necessary for haustorium differentiation or that the presence of a *P. aegyptiaca*-specific metabolite(s) prevents haustorium development.

**Figure 9:**
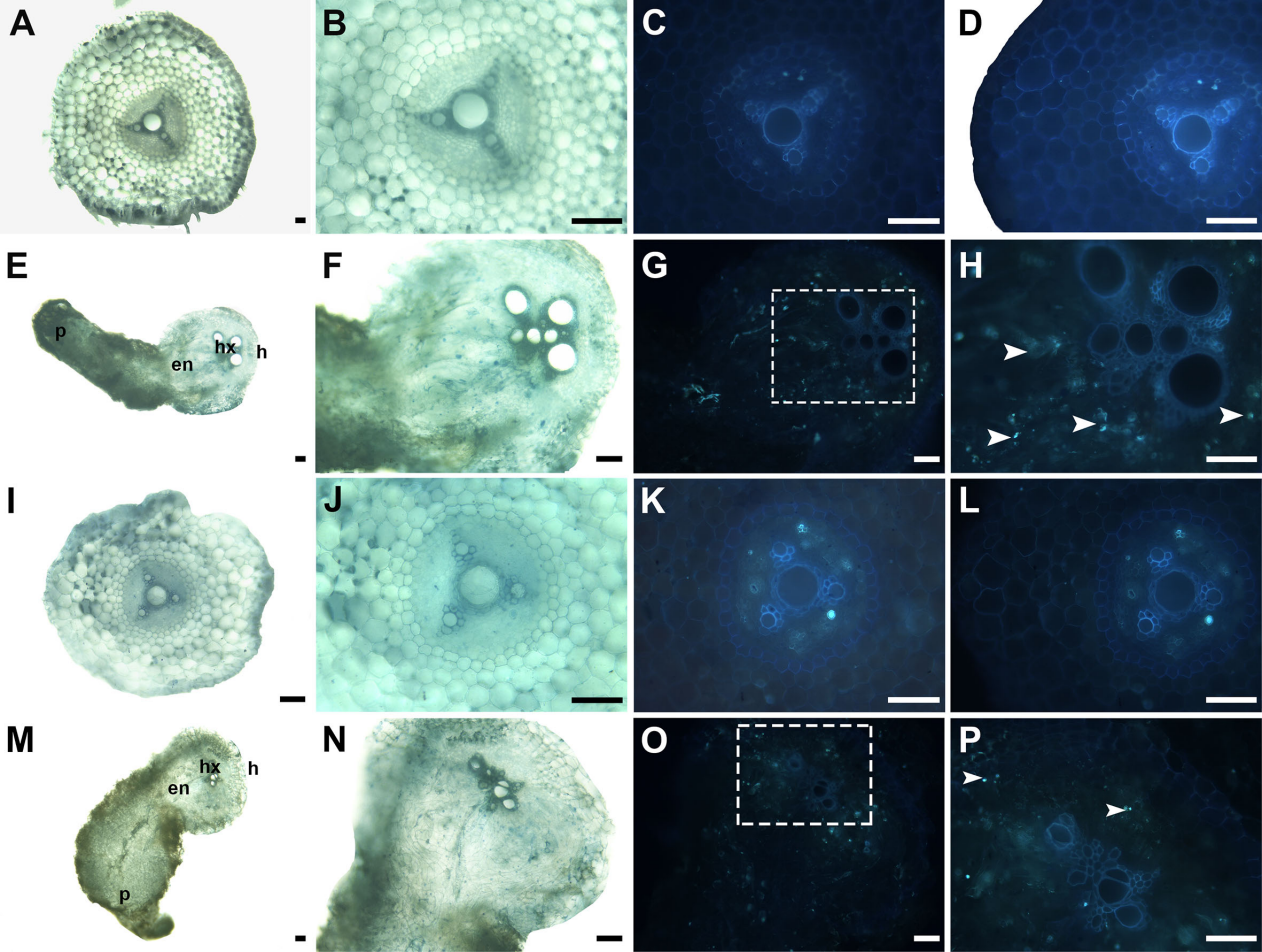
Cross-sections of incompatible and compatible interactions of *P. aegyptiaca* on KR1326 and K1076 stained with aniline blue at 16 DAI. Callose deposition shows as a blue-white fluorescence (arrows). **(A, B, E, F)** Light micrographs of fresh hand-cut sections of noninfected **(A, B)** and infected **(E, F)** with broomrape roots of KR1326. **(C, D, G, H)** The same as **(A, B, E, F)** observed under fluorescence (340–380 nm). **(H)** Magnification of the square box in **(G)**. **(I, J, M, N)** Light micrograph of a fresh hand-cut section of a noninfected **(I, J)** and infected **(M, N)** with broomrape roots of K1076. **(K, L, O, P)** The same as **(I, J, M, N)** observed under fluorescence (340–380 nm). **(P)** Magnification of the square box in O. Bars = 100 μm. h, host; p, parasite (*P. aegyptiaca*); hx, host xylem; en, endophyte.

Aniline blue fluorochrome staining was used to detect callose accumulation at the P. aegyptiaca-KR1326 resistant interaction sites (**Figures 9E–H**) and P. aegyptiaca-K1076 susceptible interaction sites (**Figures 9M–P**). The melon roots of non-infected K1076 (Figures 9I–L) and KR1326 (**Figures 9A–D**) were used as controls. Some callose deposition occurred in cells at the KR1326-parasite interface and host endodermis (**Figures 9G, H**). The resistant interaction showed stronger fluorescence than susceptible interaction (**Figures 9O, P**).

## Discussion

### Screening of melon for resistance to *P. aegyptiaca*

In many previous studies, the resistance level to *Orobanche* spp. in diverse cultivated crops was evaluated using different approaches and parameters based on the number of *Orobanche* tubercles/shoots per host plant and the impacts on yield and host development (Labrousse et al., 2001; Echevarría-Zomeño et al., 2006; Trabelsi et al., 2016; Bai et al., 2020).

In this study, a rhizotron experiment was performed to evaluate the resistance of 27 melon cultivars against *P. aegyptiaca*. Regarding the analyses of *P. aegyptiaca* resistance in the rhizotron experiment, the results showed that K986, Mibao No.1, Jingpin 2010, Xinxuelihong, K1217, Huangpi 9818, Baimei, Qingcuimi, KR1327, Xinmi No.28, and KR1326 with lower tubercles numbers were highly resistant against *P. aegyptiaca* compared to Naxigan. However, the resistance ranking by the parasitism degree and rate in the pot experiment showed that K1217, Mibao No.1, Qingcuimi, Baimei, KR1327, and Xinmi No.28 were susceptible to *P. aegyptiaca.* This inconsistency likely occurred because of the variability in broomrape sizes. For example, a melon cultivar may have multiple small-sized attachments (in tubercle stage) with postattachment resistance. This variation likely skewed the data and gave the impression that this cultivar was susceptible to broomrape. Previous studies showed that the mean number of attachments and the parasitism degree and rate did not accurately determine the resistance of host plants (Trabelsi et al., 2016; Mbuvi et al., 2017). For this reason, mean broomrape biomass seemed to be a good measure of resistance, as it considered both the number of attached parasites as well as their size. Rubiales et al. (1996) suggested that a screening based only on the number of emerged stems was misleading and that the health of the host plant must also be considered. In the pot experiment, K1217, Jingtianmi No.17, KR1326, Xuemi, Huangpi 9818, and Baimei showed better resistance. *P. aegyptiaca* had no significant adverse effects on GDW, RDW, PH, or SD in these cultivars, and the biomass of broomrape was significantly lower than in the susceptible control.

Laboratory analysis provided a unique opportunity to minimize environmental variation and ensure that the observed resistance rankings could be confidently associated with the cultivar (Mbuvi et al., 2017). However, these data did not always translate to the field due to other factors that may influence resistance through genetic χ environment interactions, which have been observed before for *Striga* and *Phelipanche ramosa* (Rodenburg and Bastiaans, 2011; Rodenburg et al., 2015; Stojanova et al., 2019). Therefore, field trials were carried out to verify the results of laboratory observations. The result was similar to the resistance rankings of melon cultivars screened in the laboratory. Field trial results showed that among seven tested cultivars (K1217, Jingtianmi No.17, KR1326, Xuemi, Huangpi 9818, Baimei, and K1076), Huangpi 9818 and KR1326 showed stable resistance levels to *P. aegyptiaca* at all field sites as expressed by a low number of emerged broomrape spikes. In addition, the yields of Huangpi 9818 and KR1326 were less affected by *P. aegyptiaca*, and the difference was not significant compared to the controls. By contrast, Xuemi and Baimei had 1.59–7.75 and 5.8–6.5 emerged broomrape spikes, significantly more than KR1326.

### KR1326 and huangpi 9818 exhibit broad-spectrum resistance against *P. aegyptiaca* populations

Our study indicates that the resistance in KR1326 and Huangpi 9818 are relatively broad spectrum and therefore potentially of great interest to farmers in the short term as well as for longer-term studies of the genetic basis of resistance for breeding. And, we found that Plot 5 *P. aegyptiaca* showed more underground attachments and biomass on KR1326 and emerged shoots on K1076 than broomrape from other populations. This indicates that the parasitic ability of Plot 5 was stronger than in *P. aegyptiaca* from other broomrape populations. However, Plot 3, 4, and 5 broomrapes were collected from processing tomatoes in Bayinytgolin Mongolia Autonomous Prefecture. In other words, variation in parasitic ability occurred even within the same broomrape ecotype. This phenomenon was similar to that observed in *Striga* (Mbuvi et al., 2017). The authors found that a *Striga* ecotype from Alupe was significantly more virulent than Mbita but was less virulent than the ecotype from Kibos, and they believed that it was difficult to associate virulence with a specific eco-geographic region. Huang et al. (2012) suggested that the high genetic diversity of the *Striga* seed bank led to rapid evolution/build-up of virulent genotypes from a subset of the natural seed bank population. However, the results of this study clearly show that there are differences in parasitic ability among the same ecotype of broomrape populations. Therefore, spatial and temporal stability and durability of *P. aegyptiaca* resistance have to be further ascertained taking into account variability in environmental conditions and virulence between *P. aegyptiaca* ecotypes.

### Evaluation of the germination-inducing activity of preattachment resistance

Previous studies have shown that low induction of germination plays a significant role in host resistance (Yoder and Scholes, 2010; Dor et al., 2011; Fernández-Aparicio et al., 2012; Trabelsi et al., 2016), but little is known about its involvement in melon response to infection by *P. aegyptiaca.* The results of our study indicated that the resistance in KR1326 was not related to low induction of *P. aegyptiaca* germination, since germination of *P. aegyptiaca* seeds was similarly stimulated by exudates of the susceptible cultivar K1076. In addition, 5-DS was detected in the roots of both cultivars, and the content was not significantly different; this result also further supports our conclusion that the resistance mechanism was not related to low induction of *P. aegyptiaca* germination. Our study did however detect differences between K1076 and KR1326 for germination induction of *O. cumana*, a broomrape species with a narrow host range that mainly parasitizes sunflowers and whose seeds germinate under low concentrations of sunflower-specific sesquiterpene lactones (Cala et al., 2017). The results showed that root exudates of K1076 induced higher levels of *O. cumana* germination in comparison with KR1326, but lower than positive control GR24. This result could explain the nonhost resistance of melon to *O. cumana* being due to low induction of germination of *O. cumana*.

### Characteristics and phenotypes of melon resistant to *P. aegyptiaca*

Different numbers of attached parasites developed successfully (compatible) or underwent necrosis (incompatible) on K1076 and KR1326. The failure of parasites to develop after attachment was associated with the appearance of broomrape tubercles darkening at the site of attachment at 23 DAI. A similar resistant phenotype was observed in legumes infected by *O. crenata* (Pérez-de-Luque et al., 2005). In this case, the tubercle necrosis reaction was associated with the lignification of host endodermis and pericycle cells at the penetration site. Cissoko et al. (2011) also showed that *Striga* grew slowly and remained small after forming only a few connections to the host xylem in the *Striga*-rice (NERICA) resistance interaction. However, this type of resistance differs from that observed in cowpea*-Striga* (Li and Timko, 2009; Su et al., 2020), sunflower-*O. cumana* (Duriez et al., 2019), or tomato-*Cuscuta* (Jhu et al., 2022). Following parasite attachment, a proportion of the parasites died owing to the onset of a rapid hypersensitive reaction between parasite and host. In our study, no clear discoloration was observed on the KR1326 roots at the parasite attachment site at 7, 9, 16, and 23 d, indicating that HR was not involved in the resistance response.

In this study, transverse sections through incompatible parasite attachments showed that the host xylem cells in contact with the parasite intrusive cells presented a thickening of their walls that stained intensely red with safranin. In addition, the safranin staining substance was also observed in the intercellular spaces between host cells and intrusive parasite cells (black triangles). Pérez-de-Luque et al. (2005) suggest that this secretion is derived from the haustorial cells of broomrape. The accumulation of secretions at the infection site may lead to the activation of xylem occlusion that may cause further necrosis of established tubercles. The accumulation of defensive secondary metabolites is an effective plant defense and competition strategy for deterring Orobanchaceae parasites (Jhu and Sinha, 2022). This observation is reminiscent of a safranin-stainable substance accumulated in the contact zone between *S. hermonthica* and *Lotus japonicus* (Yoshida and Shirasu, 2009). Lignification of endodermal cell walls in contact with parasitic tissues was observed in resistant fava beans upon *O. crenata* attack (Pérez‐de‐Luque et al., 2007). This can be explained by delayed penetration and establishment as a result of the barriers activated by the resistant host; it would take more time for the parasite to establish a functional haustorium because it must overcome the resistance mechanisms.

Callose deposition was observed in the resistance interaction at the host–parasite interface and in the middle lamellae and cell walls of some xylem vessels. This suggested that callose may be implicated in cell wall reinforcement. Reinforcement of host cell walls by callose deposition, a β-1,3-glucan polymer, appears to be the main factor responsible for resistance in this case (Pérez-de-Luque et al., 2007). In both resistant fava bean and pea plants, callose accumulation to reinforce cell walls and hamper *O. crenata* penetration was also observed (Pérez-de-Luque et al., 2006; Pérez-de-Luque et al., 2007). Because the reinforcement of cell walls by callose requires cross-linking of hydroxyproline-rich glycoproteins (Brown et al., 1998), protein cross-linking may also take place. Therefore, in subsequent experiments, the presence of protein crosslinks in the cell wall should also be investigated.

## Conclusion

Breeding of resistant cultivars is considered an essential and cost-effective component of the integrated broomrape management program. This study has shown that KR1326 exhibited very strong postattachment resistance and tolerance to *P. aegyptiaca*. The high postattachment resistance was partly attributable to the development of broomrape being arrested. Candidate melon cultivars for *P. aegyptiaca* resistance have been identified and are available for multiplication and subsequent improvement. However, the molecular mechanisms underlying this form of resistance to *P. aegyptiaca* remain unclear. Therefore, it is essential to use genetic and melon genomic techniques to “pinpoint” the resistance mechanisms that are responsible for resistance in melon. Such insights would facilitate the stacking of appropriate resistance loci in farmer-preferred and parasite-tolerant cultivars to enhance the durability and stability of defense in the long term (Scholes and Press, 2008; Rodenburg and Bastiaans, 2011).

## Data availability statement

The original contributions presented in the study are included in the article/**Supplementary Material**, further inquiries can be directed to the corresponding author/s.

## Author contributions

XC, SZ, and ZY designed the research. XC, LX, LZ, and MC performed the experiments. XC, PB, QM, and SC performed the data analysis and interpretation. XC, SZ, and ZY wrote the manuscript. All authors contributed to the article and approved the submitted version.

## Supplementary material

Supplementary Figure 1
**(A)** Map of the six sampled *P. aegyptiaca* plots in Xinjiang, China. **(B)** Phylogenetic tree of ITS and *rps2* gene constructed using maximum likelihood method. Branches with a bootstrap inferior than 60 are indicated in grey; branches with a bootstrap between 60 and 80 are indicated in yellow; branches with a bootstrap between 81 and 100 are indicated in red.

Supplementary Figure 2 The sketch of the field layout screening trial plot. **(A)** The sketch of the plot at the Majiaping screening trial in 2019 and 2021. **(B)** The sketch of the plot at the Experimental station screening trial in 2018.

Supplementary Figure 3
*P. aegyptiaca* growing on the roots of muskmelon cultivars (in rhizotrons) at 35DAI. **(A, B)** K1076 and K1237 are very susceptible, showing many *P. aegyptiaca* attachments. **(C)** Huang No.25 is susceptible, showing broomrape can develop to the “spider” stage. **(D)** Many tubercles grow on the roots of KR1328. **(E, F)** Huangpi 9818 and KR1326 exhibited good levels of resistance to the parasite with many necrotic tubercles. The black arrow indicates that broomrape can grow and develop normally, and the red arrow indicates that the tubercle is necrotic.

## References

[B1] Aly R. (2013). Trafficking of molecules between parasitic plants and their hosts. Weed. Res. 53, 231–241. doi: 10.1111/wre.12025

[B2] Anderson I. C. Cairney J. W. G. (2004). Diversity and ecology of soil fungal communities: Increased understanding through the application of molecular techniques. Environ. Microbio. 6, 769–779. doi: 10.1111/j.1462-2920.2004.00675.x 15250879

[B3] Bai J. Wei Q. Shu J. Gan Z. Li B. Yan D.. (2020). Exploration of resistance to *Phelipanche aegyptiaca* in tomato. Pest Manage. Sci. 76, 3806–3821. doi: 10.1002/ps.5932 32483849

[B4] Bao Y. Z. Yao Z. Q. Cao X. L. Peng J. F. Xu Y. Chen M. X.. (2017). Transcriptome analysis of *Phelipanche aegyptiaca* seed germination mechanisms stimulated by fluridone, TIS108, and GR24. PloS One 12, e0187539. doi: 10.1371/journal.pone.0187539 29099877 PMC5669479

[B5] Bin L. Wang C. Liu Z. He W. Zhao D. Fang Y. Y.. (2022). Geographical origin traceability of muskmelon from xinjiang province using stable isotopes and multi-elements with chemometrics. J. Food Compos. Anal. 106, 104320. doi: 10.1016/j.jfca.2021.104320

[B6] Bordallo J. Lopez-Llorca L. Jansson H. B. Salinas J. Persmark L. Asensio L. (2002). Colonization of plant roots by egg-parasitic and nematode-trapping fungi. New Phyto. 154, 491–499. doi: 10.1046/j.1469-8137.2002.00399.x 33873431

[B7] Brown I. Trethowan J. Kerry M. Mansfield J. Bolwell G. P. (1998). Localization of components of the oxidative cross-linking of glycoproteins and of callose synthesis in papillae formed during the interaction between non-pathogenic strains of *Xanthomonas campestris* and French bean mesophyll cells. Plant J. 15, 333–343. doi: 10.1046/j.1365-313X.1998.00215.x

[B8] Cala A. Molinillo J. M. Fernández-Aparicio M. Ayuso J. Álvarez J. A. Rubiales D.. (2017). Complexation of sesquiterpene lactones with cyclodextrins: Synthesis and effects on their activities on parasitic weeds. Org. Biomol. Chem. 15, 6500–6510. doi: 10.1039/c7ob01394a 28745382

[B9] Cao X. Sun C. Zhao Q. Yao Z. Zhang L. Zhao S. (2020). Indentification and evaluation for resistance of wild and cultivated melon germplasm to *Phelipanche aegyptiaca* . Acta Agricult. Boreali-occidentalis. Sin. 29, 1–9. doi: 10.7606/j.issn.1004—1389.2020.11.018

[B10] Cissoko M. Boisnard A. Rodenburg J. Press M. C. Scholes J. D. (2011). New rice for Africa (NERICA) cultivars exhibit different levels of post-attachment resistance against the parasitic weeds *Striga hermonthica* and *Striga asiatica* . New Phyto. 192, 952–963. doi: 10.1111/j.1469-8137.2011.03846.x 21883232

[B11] Díaz-Ruiz R. Torres A. M. Satovic Z. Gutierrez M. V. Cubero J. I. Román B. (2010). Validation of QTLs for *Orobanche crenata* resistance in faba bean (*Vicia faba* l.) across environments and generations. Theor. Appl. Genet. 120, 909–919. doi: 10.1007/s00122-009-1220-1 19956921

[B12] Dor E. Galili S. Smirnov E. Hacham Y. Amir R. Hershenhorn J. (2017). The effects of herbicides targeting aromatic and branched chain amino acid biosynthesis support the presence of functional pathways in broomrape. Front. Plant Sci. 8. doi: 10.3389/fpls.2017.00707 PMC5415608 28523011

[B13] Dor E. Yoneyama K. Wininger S. Kapulnik Y. Yoneyama K. Koltai H.. (2011). Strigolactone deficiency confers resistance in tomato line SL-ORT1 to the parasitic weeds *Phelipanche* and orobanche spp. Phytopathology 101, 213–222. doi: 10.1094/PHYTO-07-10-0184 20942651

[B14] Duriez P. Vautrin S. Auriac M. C. Bazerque J. Boniface M. C. Callot C.. (2019). A receptor-like kinase enhances sunflower resistance to *Orobanche cumana* . Nat. Plants. 5, 1211–1215. doi: 10.1038/s41477-019-0556-z 31819219

[B15] Echevarría-Zomeño S. Pérez-de-Luque A. Jorrín J. Maldonado A. M. (2006). Pre-haustorial resistance to broomrape (*Orobanche cumana*) in sunflower (*Helianthus annuus*): Cytochemical studies. J. Exp. Bot. 57, 4189–4200. doi: 10.1093/jxb/erl195 17095573

[B16] Eizenberg H. Goldwasser Y. (2018). Control of Egyptian broomrape in processing tomato: A summary of 20 years of research and successful implementation. Plant Dis. 102, 1477–1488. doi: 10.1094/PDIS-01-18-0020-FE 30673429

[B17] Fernández-Aparicio M. Moral A. Kharrat M. Rubiales D. (2012). Resistance against broomrapes (*Orobanche* and *Phelipanche* spp.) in faba bean (*Vicia faba*) based in low induction of broomrape seed germination. Euphytica 186, 897–905. doi: 10.1007/s10681-012-0686-0

[B18] Fernandez-Aparicio M. Sillero J. C. Rubiales D. (2009). Resistance to broomrape species (*Orobanche* spp.) in common vetch (*Vicia sativa* l.). Crop Prot. 28, 7–12. doi: 10.1016/j.cropro.2008.08.001

[B19] Fernández-Martínez J. Melero-Vara J. Muñoz-Ruz J. Ruso J. Domínguez J. (2000). Selection of wild and cultivated sunflower for resistance to a new broomrape race that overcomes resistance of the *Or_5_ * gene. Crop Sci. 40, 550–555. doi: 10.2135/cropsci2000.402550x

[B20] Furuta K. M. Xiang L. Cui S. Yoshida S. (2021). Molecular dissection of haustorium development in orobanchaceae parasitic plants. Plant Physiol. 186, 1424–1434. doi: 10.1093/plphys/kiab153 33783524 PMC8260117

[B21] Gobena D. Shimels M. Rich P. J. Ruyter-Spira C. Bouwmeester H. Kanuganti S.. (2017). Mutation in sorghum *LOW GERMINATION STIMULANT 1* alters strigolactones and causes *Striga* resistance. P. Natl. Acad. Sci. U.S.A. 114, 4471–4476. doi: 10.1073/pnas.161896511 PMC5410831 28396420

[B22] Goldwasser Y. Eizenberg H. Hershenhorn J. Plakhine D. Blumenfeld T. Buxbaum H.. (2001). Control of *Orobanche aegyptiaca* and *O. ramosa* in potato. Crop Prot. 20, 403–410. doi: 10.1016/S0261-2194(00)00162-9

[B23] González-Verdejo C. I. Fernández-Aparicio M. Córdoba E. M. López-Ráez J. A. Nadal S. (2021). Resistance against *Orobanche crenata* in bitter vetch (*Vicia ervilia*) germplasm based on reduced induction of *Orobanche* germination. Plants 10, 348. doi: 10.3390/plants10020348 33673056 PMC7917932

[B24] Goyet V. Wada S. Cui S. Wakatake T. Shirasu K. Montiel G.. (2019). Haustorium inducing factors for parasitic orobanchaceae. Front. Plant Sci. 10. doi: 10.3389/fpls.2019.01056 PMC6726735 31555315

[B25] Gurney A. Slate J. Press M. Scholes J. (2006). A novel form of resistance in rice to the angiosperm parasite *Striga hermonthica* . New Phyto. 169, 199–208. doi: 10.1111/j.1469-8137.2005.01560 16390431

[B26] Huang K. Whitlock R. Press M. C. Scholes J. D. (2012). Variation for host range within and among populations of the parasitic plant *Striga hermonthica* . Heredity 108, 96–104. doi: 10.1038/hdy.2011.52 21731054 PMC3262869

[B27] Jhu M.-Y. Farhi M. Wang L. Philbrook R. N. Belcher M. S. Nakayama H.. (2022). Heinz-Resistant tomato cultivars exhibit a lignin-based resistance to field dodder (*Cuscuta campestris*) parasitism. Plant Physiol. 189, 129–151. doi: 10.1093/plphys/kiac024 35099559 PMC9070836

[B28] Jhu M.-Y. Sinha N. R. (2022). Parasitic plants: An overview of mechanisms by which plants perceive and respond to parasites. Annu. Rev. Plant Biol. 73, 433–455. doi: 10.1146/annurev-arplant-102820-100635 35363532

[B29] Joel D. M. (2000). The long-term approach to parasitic weeds control: Manipulation of specific developmental mechanisms of the parasite. Crop Prot. 19, 753–758. doi: 10.1016/S0261-2194(00)00100-9

[B30] Kannan C. Aditi P. Zwanenburg B. (2015). Quenching the action of germination stimulants using borax and thiourea, a new method for controlling parasitic weeds: A proof of concept. Crop Prot. 70, 92–98. doi: 10.1016/j.cropro.2015.01.008

[B31] Labrousse P. Arnaud M. C. Serieys H. Bervillé A. Thalouarn P. (2001). Several mechanisms are involved in resistance of *Helianthus* to *Orobanche cumana* wallr. Ann. Bot-London. 88, 859–868. doi: 10.1006/anbo.2001.1520

[B32] Li J. Timko M. P. (2009). Gene-for-gene resistance in *Striga*-cowpea associations. Science 325, 1094–1094. doi: 10.1126/science.1174754 19713520

[B33] Mbuvi D. A. Masiga C. W. Kuria E. Masanga J. Wamalwa M. Mohamed A.. (2017). Novel sources of witchweed (*Striga*) resistance from wild sorghum accessions. Front. Plant Sci. 8. doi: 10.3389/fpls.2017.00116 PMC5292437 28220136

[B34] Motonami N. Ueno K. Nakashima H. Nomura S. Mizutani M. Takikawa H.. (2013). The bioconversion of 5-deoxystrigol to sorgomol by the sorghum, *Sorghum bicolor* (L.) moench. Phytochemistry 93, 41–48. doi: 10.1016/j.phytochem.2013.02.017 23597492

[B35] Mutuku J. M. Cui S. Hori C. Takeda Y. Tobimatsu Y. Nakabayashi R.. (2019). The structural integrity of lignin is crucial for resistance against *Striga hermonthica* parasitism in rice. Plant Physiol. 179, 1796–1809. doi: 10.1104/pp.18.01133 30670602 PMC6446757

[B36] Mutuku J. M. Cui S. Yoshida S. Shirasu K. (2021). Orobanchaceae parasite-host interactions. New Phyto. 230, 46–59. doi: 10.1111/nph.17083 33202061

[B37] Nelson D. C. (2021). The mechanism of host-induced germination in root parasitic plants. Plant Physiol. 185, 1353–1373. doi: 10.1093/plphys/kiab043 33793958 PMC8133615

[B38] Pageau K. Simier P. Le Bizec B. Robins R. J. Fer A. (2003). Characterization of nitrogen relationships between *Sorghum bicolor* and the root-hemiparasitic angiosperm *Striga hermonthica* (Del.) benth. using K^15^NO_3_ as isotopic tracer. J. Exp. Bot. 54, 789–799. doi: 10.1093/jxb/erg081 12554722

[B39] Park J.-M. Manen J.-F. Schneeweiss G. M. (2007). Horizontal gene transfer of a plastid gene in the non-photosynthetic flowering plants *Orobanche* and *Phelipanche* (Orobanchaceae). Mol. Phylogenet. Evol. 43, 974–985. doi: 10.1016/j.ympev.2006.10.011 17116411

[B40] Parker C. (2009). Observations on the current status of *Orobanche* and *Striga* problems worldwide. Pest Manage. Sci. 65, 453–459. doi: 10.1002/ps.1713 19206075

[B41] Peng J. Yao Z. Bao Y. Cao X. Chen M. Chen H. (2018). Resistance identification of different muskmelon varieties in xinjiang to *Orobanche aegyptiaca* . Xinjiang. Agric. Sci. 55, 95–104. doi: 10.6048/j.issn.1001-4330.2018.01.011

[B42] Pérez-de-Luque A. Lozano M. D. Cubero J. I. González-Melendi P. Risueño M. C. Rubiales D. (2006). Mucilage production during the incompatible interaction between *Orobanche crenata* and *Vicia sativa* . J. Exp. Bot. 57, 931–942. doi: 10.1093/jxb/erj078 16473889

[B43] Pérez-de-Luque A. Lozano M. D. Moreno M. Testillano P. Rubiales D. (2007). Resistance to broomrape (*Orobanche crenata*) in faba bean (*Vicia faba*): Cell wall changes associated with prehaustorial defensive mechanisms. Ann. Appl. Biol. 151, 89–98. doi: 10.1111/j.1744-7348.2007.00164.x

[B44] Pérez-de-Luque A. Rubiales D. Cubero J. I. Press M. Scholes J. Yoneyama K.. (2005). Interaction between *Orobanche crenata* and its host legumes: Unsuccessful haustorial penetration and necrosis of the developing parasite. Ann. Bot-London. 95, 935–942. doi: 10.1093/aob/mci105 PMC4246752 15749751

[B45] Rodenburg J. Bastiaans L. (2011). Host-plant defence against *Striga* spp.: Reconsidering the role of tolerance. Weed. Res. 51, 438–441. doi: 10.1111/j.1365-3180.2011.00871.x

[B46] Rodenburg J. Cissoko M. Kayeke J. Dieng I. Khan Z. R. Midega C. A.. (2015). Do NERICA rice cultivars express resistance to *Striga hermonthica* (Del.) benth. and *Striga asiatica* (L.) kuntze under field conditions? Field Crops Res. 170, 83–94. doi: 10.1016/j.fcr.2014.10.010 26089591 PMC4459690

[B47] Rubiales D. (2003). Parasitic plants, wild relatives and the nature of resistance. New Phyto. 160, 459–461. doi: 10.1046/j.1469-8137.2003.00929.x 33873650

[B48] Rubiales D. Flores F. Emeran A. A. Kharrat M. Amri M. Rojas-Molina M. M.. (2014). Identification and multi-environment validation of resistance against broomrapes (*Orobanche crenata* and *Orobanche foetida*) in faba bean (*Vicia faba*). Field Crops Res. 166, 58–65. doi: 10.1016/j.fcr.2014.06.010

[B49] Rubiales D. Pérez-de-Luque A. Joel D. M. Alcántara C. Sillero J. C. (2003). Characterization of resistance in chickpea to crenate broomrape (*Orobanche crenata*). Weed. Sci. 51, 702–707. doi: 10.1614/P2002-151

[B50] Rubiales D. Sillero J. Moreno M. (1996). “Preliminary screening for broomrape (*Orobanche crenata*) resistance in *Vicia* species,” in Congresos y Jornadas-Junta de Andalucia, Espana (Cordoba, Spain: JA, DGIA). Available at: https://agris.fao.org/agris-search/search.do?recordID=ES9701638.

[B51] Schneeweiss G. M. Colwell A. Park J.-M. Jang C.-G. Stuessy T. F. (2004). Phylogeny of holoparasitic *Orobanche* (Orobanchaceae) inferred from nuclear ITS sequences. Mol. Phylogenet. Evol. 30, 465–478. doi: 10.1016/S1055-7903(03)00210-0 14715236

[B52] Scholes J. D. Press M. C. (2008). *Striga* infestation of cereal crops–an unsolved problem in resource limited agriculture. Curr. Opin. Plant Biol. 11, 180–186. doi: 10.1016/j.pbi.2008.02.004 18337158

[B53] Serghini K. de Luque A. P. Castejón-Muñoz M. García-Torres L. Jorrín J. (2001). Sunflower (*Helianthus annuus* l.) response to broomrape (*Orobanche cernua* loefl.) parasitism: induced synthesis and excretion of 7-hydroxylated simple coumarins. J. Exp. Bot. 52, 2227–2234. doi: 10.1093/jexbot/52.364.2227 11604462

[B54] Stojanova B. Delourme R. Duffe P. Delavault P. Simier P. (2019). Genetic differentiation and host preference reveal non-exclusive host races in the generalist parasitic weed *Phelipanche ramosa* . Weed. Res. 59, 107–118. doi: 10.1111/wre.12353

[B55] Su C. Liu H. Wafula E. K. Honaas L. de Pamphilis C. W. Timko M. P. (2020). SHR4z, a novel decoy effector from the haustorium of the parasitic weed *Striga gesnerioides*, suppresses host plant immunity. New Phyto. 226, 891–908. doi: 10.1111/nph.16351 PMC7187149 31788811

[B56] Thorogood C. Hiscock S. (2010). Compatibility interactions at the cellular level provide the basis for host specificity in the parasitic plant *Orobanche* . New Phyto. 186, 571–575. doi: 10.1111/j.1469-8137.2009.03173.x 20522165

[B57] Trabelsi I. Abbes Z. Amri M. Kharrat M. (2016). Study of some resistance mechanisms to *Orobanche* spp. infestation in faba bean (*Vicia faba* l.) breeding lines in Tunisia. Plant Prod. Sci. 19, 562–573. doi: 10.1080/1343943X.2016.1221734

[B58] Westwood J. H. de Pamphilis C. W. Das M. Fernández-Aparicio M. Honaas L. A. Timko M. P.. (2012). The parasitic plant genome project: New tools for understanding the biology of *Orobanche* and *Striga* . Weed. Sci. 60, 295–306. doi: 10.1614/WS-D-11-00113.1

[B59] Yang C. Xu L. Zhang N. Islam F. Song W. Hu L.. (2017). iTRAQ-based proteomics of sunflower cultivars differing in resistance to parasitic weed *Orobanche cumana* . Proteomics 17, 1700009. doi: 10.1002/pmic.201700009 28618117

[B60] Yao Z. Tian F. Cao X. Xu Y. Chen M. Xiang B.. (2016). Global transcriptomic analysis reveals the mechanism of *Phelipanche aegyptiaca* seed germination. Int. J. Mol. Sci. 17, 1139. doi: 10.3390/ijms17071139 27428962 PMC4964512

[B61] Yoder J. I. Scholes J. D. (2010). Host plant resistance to parasitic weeds; recent progress and bottlenecks. Curr. Opin. Plant Biol. 13, 478–484. doi: 10.1016/j.pbi.2010.04.011 20627804

[B62] Yoneyama K. Xie X. Sekimoto H. Takeuchi Y. Ogasawara S. Akiyama K.. (2008). Strigolactones, host recognition signals for root parasitic plants and arbuscular mycorrhizal fungi, from fabaceae plants. New Phyto. 179, 484–494. doi: 10.1111/j.1469-8137.2008.02462.x 19086293

[B63] Yoshida S. Shirasu K. (2009). Multiple layers of incompatibility to the parasitic witchweed, *Striga hermonthica* . New Phyto. 183, 180–189. doi: 10.1111/j.1469-8137.2009.02840.x 19402875

[B64] Zhang X. Yao Z. Zhao S. Ding L. Du J. (2012). Distribution, harmfulness and its assessment of *Orobanche aegyptiaca* in xinjiang province. Plant Quarantine. 6, 31–39. doi: 10.19662/j.cnki.issn1005-2755.2012.06.009

