# Supplementary figures and images for: Phenotypic and histological analyses on the resistance of melon to Phelipanche aegyptiaca
Source: Front Plant Sci. 2023 Mar 24;14:1070319. doi: 10.3389/fpls.2023.1070319 (PMC10079939; doi:10.3389/fpls.2023.1070319)

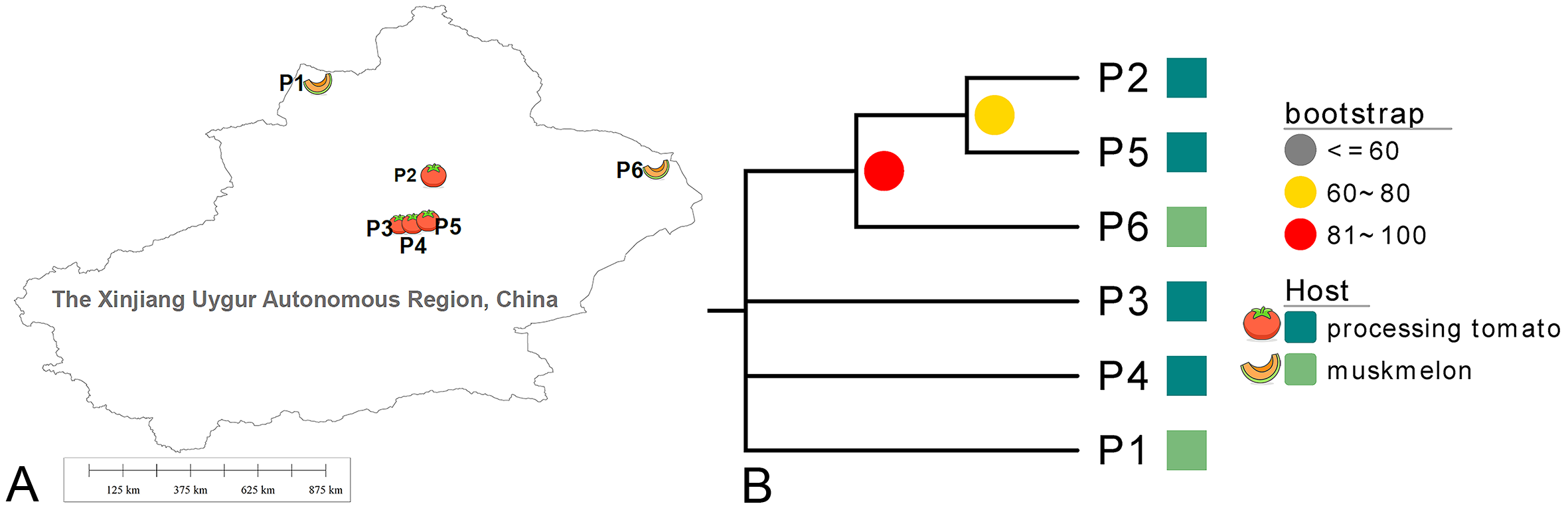

Supplement: Supplementary Figure 1 — (A) Map of the six sampled P. aegyptiaca plots in Xinjiang, China. (B) Phylogenetic tree of ITS and rps2 gene constructed using maximum likelihood method. Branches with a bootstrap inferior than 60 are indicated in grey; branches with a bootstrap between 60 and 80 are indicated in yellow; branches with a bootstrap between 81 and 100 are indicated in red. [file Image_1.tif]

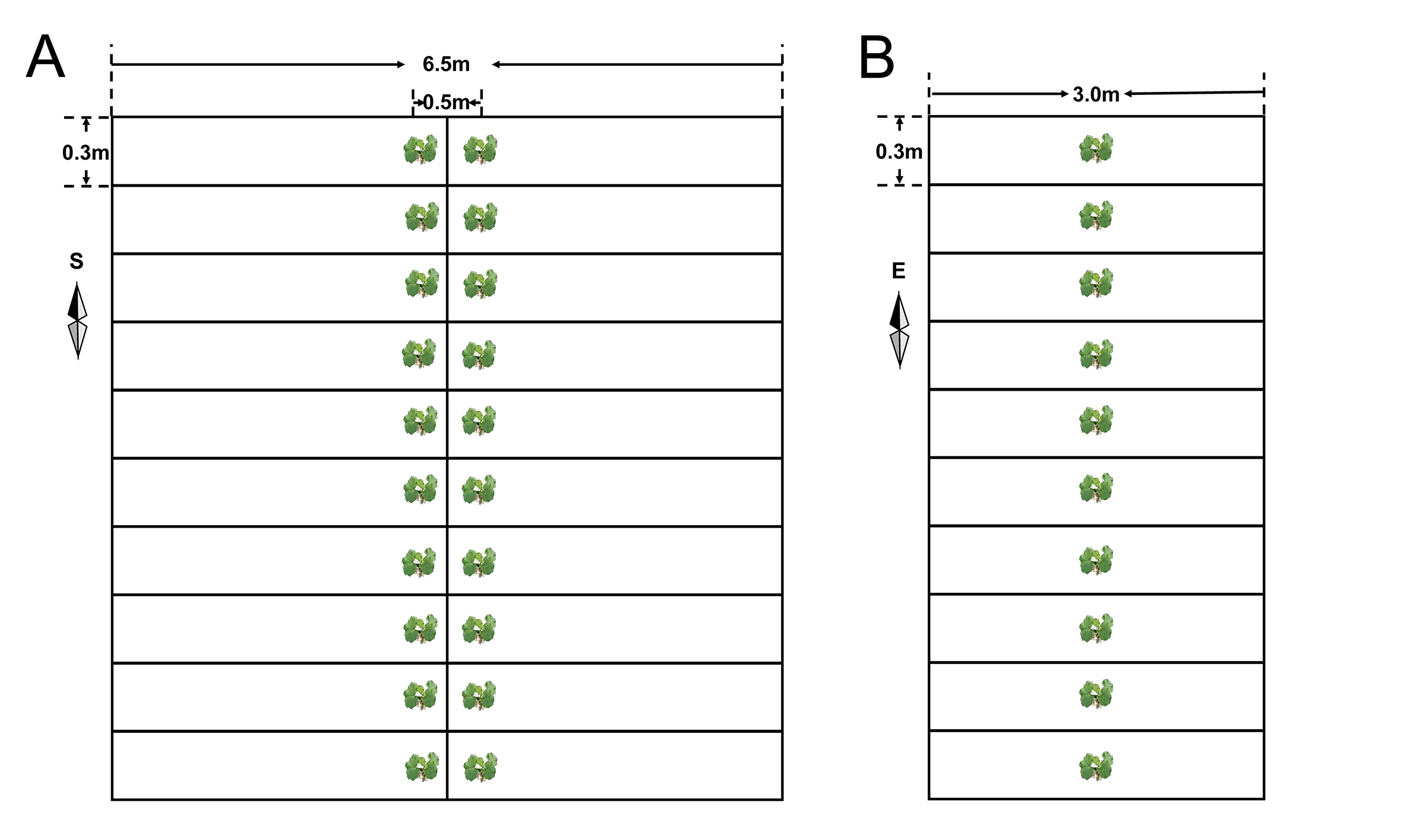

Supplement: Supplementary Figure 2 — The sketch of the field layout screening trial plot. (A) The sketch of the plot at the Majiaping screening trial in 2019 and 2021. (B) The sketch of the plot at the Experimental station screening trial in 2018. [file Image_2.tif]

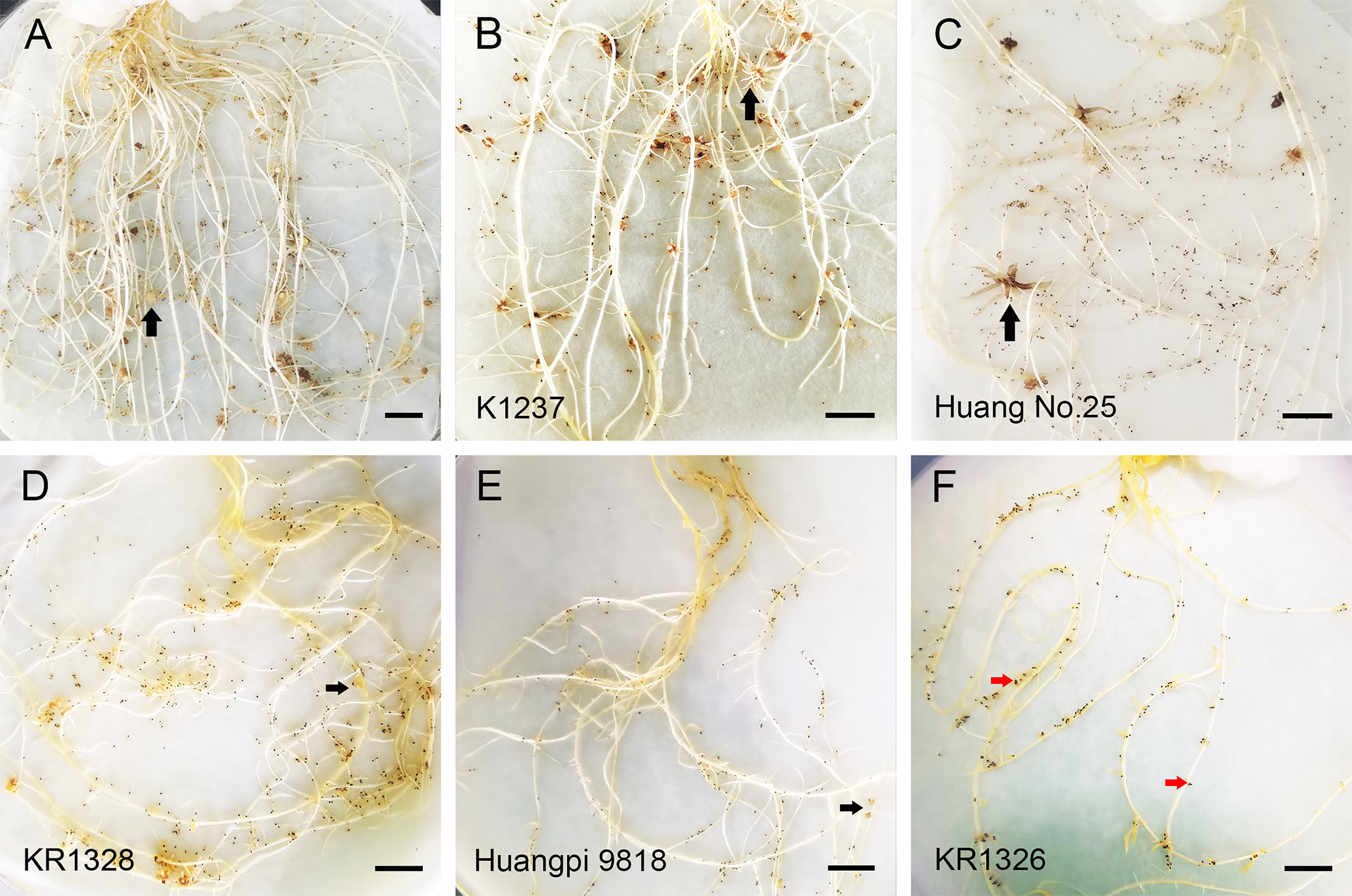

Supplement: Supplementary Figure 3 — P. aegyptiaca growing on the roots of muskmelon cultivars (in rhizotrons) at 35DAI. (A, B) K1076 and K1237 are very susceptible, showing many P. aegyptiaca attachments. (C) Huang No.25 is susceptible, showing broomrape can develop to the “spider” stage. (D) Many tubercles grow on the roots of KR1328. (E, F) Huangpi 9818 and KR1326 exhibited good levels of resistance to the parasite with many necrotic tubercles. The black arrow indicates that broomrape can grow and develop normally, and the red arrow indicates that the tubercle is necrotic. [file Image_3.tif]
