# Supplementary material for: Phenotypic and histological analyses on the resistance of melon to Phelipanche aegyptiaca
Source: Front Plant Sci. 2023 Mar 24;14:1070319. doi: 10.3389/fpls.2023.1070319 (PMC10079939; doi:10.3389/fpls.2023.1070319)
Supplement: Supplementary file 4 [file Table_1.docx]

Supplementary Table 1 Details of the melon cultivars used in this study

| Cultivar name | Type | Origin | Seed source |
| --- | --- | --- | --- |
| Naxigan | Thick peel | Changji, Xinjiang, China | XJYF |
| Huang No.25 | Thick peel | Hami, Xinjiang, China | XJNB |
| Jingpin 2010 | Thick peel | Hami, Xinjiang, China | XJNB |
| Jingpinxiaoxiangfei | Thick peel | Hami, Xinjiang, China | XJNB |
| Jingtianmi No.17 | Thick peel | Hami, Xinjiang, China | XJNB |
| Qinghuami | Thick peel | Hami, Xinjiang, China | XJNB |
| Mibao No.1 | Thick peel | Hami, Xinjiang, China | XJNB |
| Tianmicui | Thick peel | Hami, Xinjiang, China | XJNB |
| Qingcuimi | Thick peel | Hami, Xinjiang, China | XJNB |
| K1386 | Thick peel | Xinjiang, China | XAAS |
| KR1327 | Thick peel | Xinjiang, China | XAAS |
| K1238 | Thick peel | Xinjiang, China | XAAS |
| K1526 | Thick peel | Xinjiang, China | XAAS |
| K1217 | Thick peel | Xinjiang, China | XAAS |
| KR1222 | Thick peel | Xinjiang, China | XAAS |
| KR1328 | Thick peel | Xinjiang, China | XAAS |
| KR1326 | Thick peel | Xinjiang, China | XAAS |
| K1237 | Thick peel | Xinjiang, China | XAAS |
| K1076 | Thick peel | Xinjiang, China | XAAS |
| K986 | Thick peel | Xinjiang, China | XAAS |
| Xuemi | Thick peel | Xinjiang, China | XAAS |
| Huangpi 9818 | Thick peel | Xinjiang, China | XAAS |
| Baimei | Thick peel | Xinjiang, China | XAAS |
| Xinmi 28 | Thick peel | Xinjiang, China | XAAS |
| Huangfei | Thick peel | Xinjiang, China | XAAS |
| Xinxuelihong | Thick peel | Xinjiang, China | XAAS |
| Fengwei No.8 | Thick peel | Xinjiang, China | XAAS |

Note: XAAS, Melon Research Center, Xinjiang Academy of Agricultural Sciences; XJYF, Xinjiang Changji Yifeng seedling Co., Ltd; XJNB, Xinjiang Nongbang Seedling Technology Development Co., Ltd.
