# Supplementary material for: Phenotypic and histological analyses on the resistance of melon to Phelipanche aegyptiaca
Source: Front Plant Sci. 2023 Mar 24;14:1070319. doi: 10.3389/fpls.2023.1070319 (PMC10079939; doi:10.3389/fpls.2023.1070319)
Supplement: Supplementary file 5 [file Table_2.docx]

Supplementary Table 2 Details of the seeds of *P. aegyptiaca* and *O. cumana* used in this study.

| Population name | Details | Latitude: longitude |
| --- | --- | --- |
| Plot 1 | Collected from melon growing in 163 Regiment, Tacheng, Xinjiang, China in 2017 | 82° 55' 10" E, 46° 47' 59" N |
| Plot 2 | Collected from processing tomato growing in Junhu farm, Changji, Xinjiang, China in 2017 | 87° 0'19" E, 44° 1' 27" N |
| Plot 3 | Collected from processing tomato growing in 21 Regiment, Bayinytgolin Mongolia Autonomous Prefecture, Xinjiang, China in 2017 | 86° 18' 54" E, 42° 9' 36" N |
| Plot 4 | Collected from processing tomato in 22 Regiment, Bayinytgolin Mongolia Autonomous Prefecture, Xinjiang, China in 2017 | 86° 33' 5" E, 42° 10' 40" N |
| Plot 5 | Collected from processing tomato growing in 25 Regiment, Bayinytgolin Mongolia Autonomous Prefecture, Xinjiang, China in 2017 | 86° 40' 36" E, 41° 59' 25" N |
| Plot 6 | Collected from melon growing in Naomaohu farm, Hami, Xinjiang, China in 2017 | 94° 58' 56" E, 43° 55' 52" N |
