# Supplementary material for: Phenotypic and histological analyses on the resistance of melon to Phelipanche aegyptiaca
Source: Front Plant Sci. 2023 Mar 24;14:1070319. doi: 10.3389/fpls.2023.1070319 (PMC10079939; doi:10.3389/fpls.2023.1070319)
Supplement: Supplementary file 6 [file Table_3.docx]

Supplementary Table 3 Identification standards of *P. aegyptiaca* resistance levels for melon

| Resistance level | Parasitism rate/% | Parasitic degree |
| --- | --- | --- |
| Immunity | 0 | 0 |
| High resistance | 0 < F ≤ 35 | 0 < AD < 1 |
| Resistance | F ≤ 75 | AD ≤ 10 |
| Susceptibility | 75 < F ≤ 100 | AD ≤ 10 |
| High susceptibility | 35 < F ≤ 100 | AD > 10 |

Note: F, Parasitism rate; AD, Parasitic degree.
