# Supplementary material for: Phenotypic and histological analyses on the resistance of melon to Phelipanche aegyptiaca
Source: Front Plant Sci. 2023 Mar 24;14:1070319. doi: 10.3389/fpls.2023.1070319 (PMC10079939; doi:10.3389/fpls.2023.1070319)
Supplement: Supplementary file 7 [file Table_4.docx]

Supplementary Table 4 Overview of experimental conditions of the field trials conducted at the experimental station (2018) and Majiaping (2019 and 2021)

| Location | Experimental station  (86° 3' 25" E, 44° 18' 44" N) | Majiaping  (86° 7' 11" E, 44° 16' 23" N) | |
| --- | --- | --- | --- |
| Altitude (m a.s.l.) | 465 | 464 | |
| Year | 2018 | 2019 | 2021 |
| Period | May–Aug | May–Aug | May–Aug |
| Sowing dates | 29/05/2018 | 14/05/2019 | 04/05/2021 |
| Net plot size (m^2^) | 200.4 m^2^ | 490 m^2^ | 490 m^2^ |
| Net sub-plot size (m^2^) | 9 m^2^ | 19.5m^2^ | 19.5m^2^ |
| Soil parameters |  |  |  |
| N(g/kg) | 0.88 | 0.88 | - |
| P(mg/Kg) | 18.47 | 11.37 | - |
| K(mg/Kg) | 179.33 | 163.67 | - |
