# Supplementary material for: Phenotypic and histological analyses on the resistance of melon to Phelipanche aegyptiaca
Source: Front Plant Sci. 2023 Mar 24;14:1070319. doi: 10.3389/fpls.2023.1070319 (PMC10079939; doi:10.3389/fpls.2023.1070319)
Supplement: Supplementary file 8 [file Table_5.docx]

Supplementary Table 5 Attachments number and biomass of *P. aegyptiaca* in the pot experiment in 2018.

| Cultivar name | Number of *P. aegyptiaca* at different growth stage/Plant | | | | Attachment dry weight (g) |
| --- | --- | --- | --- | --- | --- |
|  | S4 | S5 | S6 | S7 |  |
| Huang No.25 | 3.33±2.03 ab (18.81) | 7.67±1.67 b (51.64) | 3.67±0.88 ab (25.13) | 0.67±0.33 a (4.42) | 0.91±0.27 a |
| K1237 | 2.40±1.25 b (11.45) | 7.40±1.96 b (44.72) | 8.20±2.82 a (43.83) | 0.00±0.00 b (0.00) | 0.37±0.03 b |
| Huangfei | 5.67±3.28 ab (23.39) | 4.17±3.23 b (18.29) | 3.83±0.54 ab (54.62) | 0.33±0.33 ab (3.70) | 0.29±0.06 bc |
| K1076 | 1.00±0.58 b (4.71) | 9.25±1.65 b (71.32) | 4.25±1.97 ab (23.97) | 0.00±0.00 b (0.00) | 0.26±0.04 bcd |
| Naxigan | 0.20±0.20 b (0.91) | 4.40±1.89 b (68.81) | 2.40±1.91 b (16.95) | 0.40±0.40 ab (13.33) | 0.21±0.00 bcde |
| KR1222 | 3.29±1.15 ab (9.10) | 31.57±4.22 a (80.78) | 3.57±0.57 b (10.12) | 0.00±0.00 b (0.00) | 0.20±0.06 cde |
| Xinxuelihong | 1.83±0.48 b (41.03) | 3.33±1.05 b (47.88) | 0.67±0.49 b (6.85) | 0.33±0.21 ab (4.23) | 0.19±0.09 cdef |
| Jingpinxiaoxiangfei | 1.75±0.90 b (9.54) | 9.38±1.58 b (72.44) | 3.13±1.01 b (18.02) | 0.00±0.00 b (0.00) | 0.16±0.04 cdef |
| K1386 | 4.50±0.56 ab (63.08) | 4.33±2.32 b (26.65) | 1.17±0.79 b (7.49) | 0.17±0.17 ab (2.78) | 0.09±0.04 def |
| Tianmicui | 3.75±1.80 a (52.65) | 1.25±0.95 b (25.00) | 2.25±1.93 b (22.35) | 0.00±0.00 b (0.00) | 0.08±0.02 ef |
| Xinmi No.28 | 1.50±0.96 b (63.03) | 0.50±0.27 b (21.01) | 0.38±0.26 b (15.97) | 0.00±0.00 b (0.00) | 0.06±0.04 ef |
| Fengwei No.8 | 1.00±1.00 b (29.94) | 1.67±1.67 b (50.00) | 0.67±0.67 b (20.06) | 0.00±0.00 b (0.00) | 0.05±0.05 ef |
| K986 | 0.86±0.40 b (35.25) | 1.29±0.75 b (52.87) | 0.29±0.29 b (11.88) | 0.00±0.00 b (0.00) | 0.04±0.02 ef |
| KR1327 | 4.00±1.03 ab (40.65) | 5.67±0.92 b (57.62) | 0.17±0.17 b (1.73) | 0.00±0.00 b (0.00) | 0.02±0.02 f |
| Qingcuimi | 1.17±0.65 b (20.63) | 4.33±2.29 b (76.37) | 0.17±0.17 b (3.00) | 0.00±0.00 b (0.00) | 0.01±0.01 f |
| K1526 | 10.33±1.99 a (63.72) | 6.33±1.91 b (36.28) | 0.00±0.00 b (0.00) | 0.00±0.00 b (0.00) | 0.01±0.00 f |
| Jingpin 2010 | 5.33±1.45 ab (46.12) | 4.33±0.99 b (53.88) | 0.00±0.00 b (0.00) | 0.00±0.00 b (0.00) | 0.01±0.00 f |
| KR1328 | 4.33±3.84 ab (61.86) | 2.67±1.76 b (38.14) | 0.00±0.00 b (0.00) | 0.00±0.00 b (0.00) | 0.01±0.00 f |
| K1238 | 3.00±1.10 ab (62.50) | 1.20±0.97 b (25.00) | 0.60±0.60 b (12.50) | 0.00±0.00 b (0.00) | 0.00±0.00 f |
| Huangpi 9818 | 0.43±0.30 b (50.00) | 0.43± 0.30 b (50.00) | 0.00±0.00 b (0.00) | 0.00±0.00 b (0.00) | 0.00±0.00 f |
| Mibao No.1 | 4.00±0.45 ab (83.61) | 0.83±0.40 b (16.39) | 0.00±0.00 b (0.00) | 0.00±0.00 b (0.00) | 0.00±0.00 f |
| Qinghuami | 3.00±0.89 ab (57.36) | 1.83±0.31 b (42.64) | 0.00±0.00 b (0.00) | 0.00±0.00 b (0.00) | 0.00±0.00 f |
| Baimei | 5.50±1.55 ab (95.00) | 0.25±0.25 b (5.00) | 0.00±0.00 b (0.00) | 0.00±0.00 b (0.00) | 0.00±0.00 f |
| Xuemi | 5.00±1.48 ab (100.00) | 0.00±0.00 b (0.00) | 0.00±0.00 b (0.00) | 0.00±0.00 b (0.00) | 0.00±0.00 f |
| Jingtianmi No.17 | 4.00±1.64 ab (96.67) | 0.20±0.20 b (3.33) | 0.00±0.00 b (0.00) | 0.00±0.00 b (0.00) | 0.00±0.00 f |
| K1217 | 3.00±2.08 ab (90.09) | 0.33±0.33 b (9.91) | 0.00±0.00 b (0.00) | 0.00±0.00 b (0.00) | 0.00±0.00 f |
| KR1326 | 0.50±0.27 b (100.00) | 0.00±0.00 b (0.00) | 0.00±0.00 b (0.00) | 0.00±0.00 b (0.00) | 0.00±0.00 f |

Note: Data are mean ± standard error of 3~7 replications. Data with the same letter per column are not significantly different according to Tukey’s HSD (*p* ≤ 0.05). The numbers in brackets indicate the percentage of broomrape at different developmental stages (%)
