# Supplementary material for: Phenotypic and histological analyses on the resistance of melon to Phelipanche aegyptiaca
Source: Front Plant Sci. 2023 Mar 24;14:1070319. doi: 10.3389/fpls.2023.1070319 (PMC10079939; doi:10.3389/fpls.2023.1070319)
Supplement: Supplementary file 9 [file Table_6.docx]

Supplementary Table 6 Effects of *P. aegyptiaca* on muskmelon total above-ground parts dry weight, root dry weight, plant height, and stem diameter in the pot experiment in 2018.

| Cultivar name | Total above-ground parts dry weight (g) | | Root dry weight per plant (g) | | Plant height (cm) | | Stem diameter (mm) | |
| --- | --- | --- | --- | --- | --- | --- | --- | --- |
|  | *P. aegyptiaca*-infested | *P. aegyptiaca*-free | *P. aegyptiaca*-infested | *P. aegyptiaca*-free | *P. aegyptiaca*-infested | *P. aegyptiaca*-free | *P. aegyptiaca*-infested | *P. aegyptiaca*-free |
| KR1222 | 0.28±0.02 | 0.50±0.01*** | 0.45±0.14 | 0.70±0.02*** | 10.17±0.48 | 20.17±1.14*** | 2.76±0.14 | 3.01±0.05NS |
| K986 | 0.41±0.03 | 0.58±0.02*** | 0.07±0.01 | 0.09±0.00* | 25.21±1.33 | 36.50±2.17** | 3.40±0.12 | 3.47±0.07NS |
| K1076 | 0.72±0.03 | 0.92±0.02** | 0.11±0.05 | 0.43±0.04** | 34.71±3.91 | 50.00±2.22** | 3.03±0.29 | 3.42±0.15NS |
| Huangfei | 0.21±0.05 | 0.43±0.04** | 0.05±0.01 | 0.25±0.03** | 14.42±0.98 | 20.75±1.44** | 3.52±0.19 | 4.10±0.42NS |
| K1237 | 0.57±0.02 | 0.67±0.01** | 0.08±0.03 | 0.21±0.05** | 21.13±1.86 | 25.50±0.43* | 3.17±0.23 | 3.60±0.09NS |
| Naxigan | 0.21±0.01 | 0.29±0.01** | 0.09±0.01 | 0.11±0.01+ | 13.00±1.71 | 19.67±1.73* | 2.59±0.26 | 3.55±0.16** |
| Jingpinxiaoxiangfei | 0.62±0.05 | 0.88±0.12* | 0.14±0.03 | 0.31±0.04** | 27.33±1.93 | 50.00±3.60*** | 2.75±0.13 | .88±0.16NS |
| Huang No.25 | 0.54±0.03 | 0.85±0.06* | 0.24±0.06 | 0.48±0.03** | 18.00±0.89 | 26.50±1.55** | 3.43±0.09 | 3.84±0.13 * |
| K1386 | 0.55±0.03 | 0.70±0.03* | 0.15±0.03 | 0.34±0.04** | 26.80±2.03 | 28.75±0.48 NS | 2.73±0.09 | 3.00±0.21NS |
| Xinxuelihong | 1.14±0.10 | 1.25±0.33NS | 0.19±0.03 | 0.35±0.03** | 22.44±1.26 | 27.25±0.48** | 3.38±0.12 | 3.66±0.12NS |
| Fengwei No.8 | 1.35±0.24 | 1.65±0.22NS | 0.11±0.02 | 0.24±0.03** | 45.17±1.19 | 50.00±1.08* | 2.75±0.11 | 2.74±0.17NS |
| Xinmi No.28 | 0.61±0.04 | 0.57±0.07NS | 0.16±0.02 | 0.29±0.03* | 16.82±1.06 | 28.75±2.06** | 3.43±0.08 | 3.52±0.09NS |
| K1526 | 0.52±0.05 | 0.66±0.13NS | 0.18±0.02 | 0.32±0.04* | 21.45±1.22 | 29.00±2.04* | 2.98±0.09 | 3.07±0.08NS |
| Qinghuami | 0.39±0.04 | 0.43±0.04NS | 0.05±0.01 | 0.07±0.00* | 13.83±0.42 | 14.50±0.29NS | 2.63±0.08 | 2.70±0.08NS |
| Mibao No.1 | 0.58±0.03 | 0.61±0.02NS | 0.07±0.00 | 0.11±0.01* | 30.92±1.55 | 33.25±1.80NS | 3.05±0.05 | 3.06±0.06NS |
| K1238 | 0.51±0.04 | 0.60±0.03NS | 0.21±0.06 | 0.21±0.03NS | 22.20±1.11 | 28.25±0.63*** | 3.03±0.11 | 3.05±0.10NS |
| K1217 | 1.19±0.19 | 1.20±0.05NS | 0.74±0.21 | 0.76±0.03NS | 16.50±1.26 | 29.75±2.66** | 2.95±0.09 | 3.07±0.04NS |
| KR1328 | 0.97±0.07 | 1.04±0.13NS | 0.31±0.05 | 0.28±0.10NS | 33.17±2.29 | 39.75±0.85* | 2.76±0.13 | 2.93±0.03NS |
| Jingtianmi No.17 | 0.50±0.05 | 0.56±0.03NS | 0.06±0.00 | 0.05±0.00NS | 20.67±1.46 | 29.00±2.68 * | 2.71±0.10 | 2.67±0.17NS |
| Qingcuimi | 0.74±0.11 | 1.01±0.38NS | 0.22±0.03 | 0.30±0.08NS | 25.08±1.32 | 27.00±3.34NS | 2.57±0.06 | 3.03±0.15NS |
| Jingpin 2010 | 0.69±0.05 | 0.81±0.12NS | 0.26±0.03 | 0.34±0.04NS | 37.25±2.37 | 39.25±1.25NS | 2.82±0.09 | 2.87±0.20NS |
| Tianmicui | 1.06±0.08 | 0.94±0.05NS | 0.15±0.02 | 0.15±0.01NS | 44.25±1.92 | 48.00±1.47NS | 3.22±0.12 | 3.69±0.22NS |
| KR1327 | 1.28±0.13 | 1.34±0.10NS | 0.13±0.13 | 0.14±0.14NS | 38.83±2.17 | 43.50±2.06NS | 2.88±0.08 | 2.91±0.13NS |
| KR1326 | 0.48±0.02 | 0.46±0.02NS | 0.23±0.05 | 0.21±0.04NS | 24.17±1.50 | 28.50±2.96NS | 3.29±0.05 | 3.15±0.15NS |
| Xuemi | 0.86±0.03 | 0.90±0.03NS | 0.21±0.03 | 0.21±0.01NS | 20.67±1.30 | 20.67±0.33NS | 3.55±0.06 | 3.53±0.06NS |
| Huangpi 9818 | 0.48±0.05 | 0.47±0.02NS | 0.09±0.02 | 0.12±0.02NS | 27.69±3.41 | 26.25±0.85NS | 3.55±0.06 | 3.60±0.10NS |
| Baimei | 1.29±0.14 | 1.13±0.15NS | 0.26±0.05 | 0.34±0.05NS | 54.50±3.44 | 55.75±0.25NS | 3.48±0.15 | 3.41±0.42NS |

Note: Data are mean ± standard error of 3~7 replications. * and **indicate significant differences at *p*< 0.05 and 0.01, respectively; *** indicates significant differences at *p*< 0.001; + indicates significant differences at *p*< 0.10, by Student t-test within a variety. NS indicates not significant (*p*> 0.10) within a variety.
